# Polyclonal Antibody Production for Membrane Proteins *via* Genetic Immunization

**DOI:** 10.1038/srep21925

**Published:** 2016-02-24

**Authors:** Debra T. Hansen, Mark D. Robida, Felicia M. Craciunescu, Andrey V. Loskutov, Katerina Dörner, John-Charles Rodenberry, Xiao Wang, Tien L. Olson, Hetal Patel, Petra Fromme, Kathryn F. Sykes

**Affiliations:** 1 Center for Innovations in Medicine, Biodesign Institute, Arizona State University, Tempe, Arizona, USA; 2 Center for Applied Structural Discovery, Biodesign Institute, Arizona State University, Tempe, Arizona, USA; 3 School of Molecular Sciences (formerly the Department of Chemistry and Biochemistry), Arizona State University, Tempe, Arizona, USA; 4 U.S. National Institute of General Medical Sciences PSI:Biology Center for Membrane Proteins in Infectious Diseases.

## Abstract

Antibodies are essential for structural determinations and functional studies of membrane proteins, but antibody generation is limited by the availability of properly-folded and purified antigen. We describe the first application of genetic immunization to a structurally diverse set of membrane proteins to show that immunization of mice with DNA alone produced antibodies against 71% (n = 17) of the bacterial and viral targets. Antibody production correlated with prior reports of target immunogenicity in host organisms, underscoring the efficiency of this DNA-gold micronanoplex approach. To generate each antigen for antibody characterization, we also developed a simple *in vitro* membrane protein expression and capture method. Antibody specificity was demonstrated upon identifying, for the first time, membrane-directed heterologous expression of the native sequences of the FopA and FTT1525 virulence determinants from the select agent *Francisella tularensis* SCHU S4. These approaches will accelerate future structural and functional investigations of therapeutically-relevant membrane proteins.

Membrane proteins are the molecular interface between host and pathogen, yet these key proteins provide unique challenges for structural elucidation^1^ and for their use in developing not only therapeutics^2^ but also diagnostics and vaccines. Target-specific monoclonal antibodies have allowed the determination of novel membrane protein structures during electron cryomicroscopy by increasing the size of the target^3^, and during crystallography^4^ by stabilizing unique protein conformations^5^,^6^,^7^,^8^, providing crystal lattice contacts^9^,^10^,^11^, and allowing structure solution *via* molecular replacement^10^,^11^,^12^. Monoclonal antibodies that recognize specific conformations of membrane-embedded signal pathway proteins allow the exciting development of novel therapeutics^13^. Towards antibody production for membrane proteins, there is often a limitation in the availability of highly-purified or natively-folded target antigen. Therefore, we explored the genetic immunization approach^14^ in order to generate antibodies that target membrane proteins.

Surprisingly, the efficiency of genetic immunization as applied to membrane proteins is unknown, since application of this method has been described only or for collections of soluble proteins^15^ or for individual membrane protein targets^16^,^17^,^18^. For these individual membrane proteins, the reported operational serum dilutions of ≤1:200 for the human thyrotropin and neurokinin-1 GPCRs^16^,^17^ and for human nephrin^18^ suggest room for improvement. The biolistic approach, using only genes as the source of antigen, has generated monoclonal antibodies that recognize native epitopes of membrane proteins^17^,^18^, including modifications such as glycosylation^18^. Additional groups have used genetic immunization alone to generate antibodies with therapeutic potential, albeit using proprietary methods^19^,^20^.

Here we describe an efficient approach that yielded antibodies against the majority of 17 membrane proteins from Biosafety Level 3 pathogens. The SCHU S4 isolate of *Francisella tularensis* is one of the most pathogenic bacteria known due to its capacity for fatal infection from as few as ten cells^21^. *F. tularensis* causes the disease tularemia and is a model intracellular bacterial pathogen given its capacity to evade the immune response and to infect numerous cell types^22^. The arthropod-borne African swine fever virus (ASFV) causes an untreatable, highly-lethal hemorrhagic porcine disease that is an economic threat in Africa and eastern Europe^23^. The endemic existence of both these pathogens throughout numerous environmental sources makes eradication implausible^24^,^25^. Investigations with endogenous protein from these organisms are constrained by biosafety requirements and select agent status. To support studies of membrane proteins that are important in pathogenesis, we developed DNA-based approaches to generate and characterize antibodies against a set of membrane proteins (Supplementary Table 1) that were targeted for structural studies as part of the U.S. National Institutes of Health’s Protein Structure Initiative (PSI:Biology). Many of these targets are expected to provide novel membrane protein structures as they lack obvious sequence homologs outside of the *Francisella* genus and the *Asfarviridae* virus family.

## Results and Discussion

### *In vitro* expression and purification of membrane proteins by IVT-HMB

**Figure 1:**
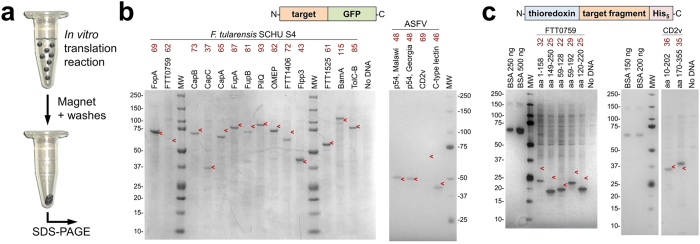
*In vitro* generation of purified membrane protein antigen. (**a**) Schematic of the IVT-HMB method for expression and purification of membrane proteins. (**b**) Generation of full-length proteins for 15 targets in the form of fusions to green fluorescent protein (GFP). (**c**) Generation of partial-length proteins for *F. tularensis* FTT0759 (305 amino acids) and ASFV CD2v (360 amino acids) in the form of fusions to thioredoxin. Shown are SDS-PAGE visualized by Coomassie stain (left panel of **b**) or silver stain (remaining panels in **b**,**c**) and containing, per lane, magnetic-bead purified fractions from 10 μL of IVT-HMB. Molecular weight (MW) in kDa is indicated, and arrowheads indicate the predicted migration position. BSA, bovine serum albumin.

To facilitate analyses of the sera, we developed a novel *in vitro* approach for simultaneous expression and capture of each of the membrane protein targets. We optimized a commercially available *in vitro* protein translation system and included unmodified tosylactivated magnetic beads in the reaction to yield the method IVT-HMB (*in vitro* translation in the presence of hydrophobic magnetic beads; Fig. 1a). Tosylated beads are typically used to capture protein via covalent modification of the bead surface through replacement of the tosyl leaving group with a capturing ligand. Rather, we found that unmodified beads allowed non-covalent capture of each of the 17 membrane protein targets at high purity (Fig. 1b,c). Using IVT-HMB, we obtained 15 of the 17 targets in full-length form (Fig. 1b). The remaining two targets were captured in multiple truncated forms that in total represented most of the length of each target sequence (Fig. 1c). The capture process may be based on bead surface adherence to hydrophobic residues that are normally buried in the membrane or that lie internal to the protein’s tertiary structure, but are exposed during the *in vitro* synthesis events.

Towards characterization of polyclonal sera, the IVT-HMB approach effectively simplified antigen preparation by precluding the need to use endogenous protein or to purify detergent-solubilized or urea-denatured membrane protein, since the protein can be directly used without a separate elution step. Although the IVT-HMB protein is not expected to be natively-folded, as GFP fluorescence of the bead-bound protein was not detectable above empty vector controls, this protein is suitable for evaluation of polyclonal immune responses since a significant proportion of polyclonal antibody species recognizes linear epitopes^26^. The resulting yields of 5–20 μg of membrane protein per 500 μL of IVT-HMB reaction were sufficient to allow completion of ELISA and Western analyses of the sera from 5 mice. Other unique advantages of the method include eliminating any need for tagging the target protein or including a capturing ligand on the beads. Limitations of the current IVT-HMB method may be in identifying conformationally-specific monoclonal antibodies and antibodies for antigens that are highly modified post-translationally. However, we expect that IVT-HMB can be adapted to *in vitro* systems that provide such modifications^27^.

### Antibody production by genetic immunization using DNA-gold micronanoplexes

**Figure 2:**
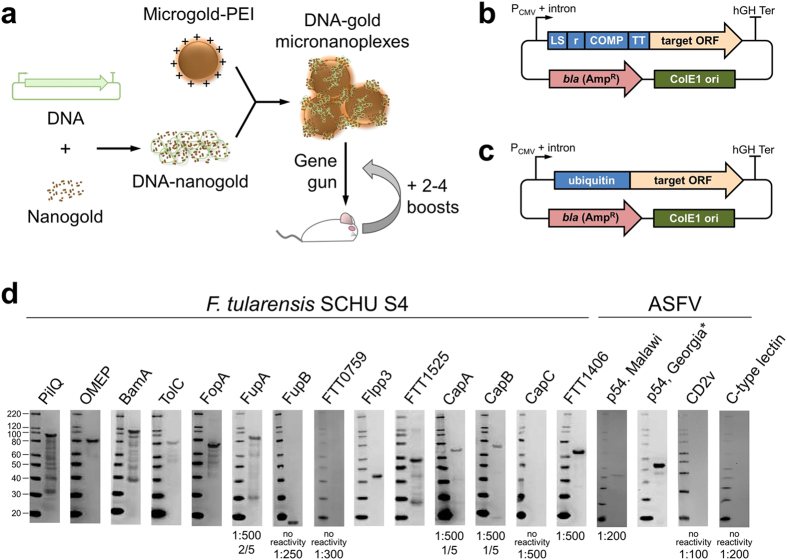
Production of high-specificity polyclonal antibodies for membrane proteins from *F. tularensis* and ASFV. (**a**) Overview of DNA-gold micronanoplex bullet production. (**b**,**c**) Genetic immunization vectors pCMVi-LSrCOMPTT (**b**) and pCMVi-UB^35^ (**c**). (**d**) Representative Westerns on IVT-HMB target protein using immunizations with pCMVi-LSrCOMPTT, except for (*) which used pCMVi-UB. Positive immunoblots were for 5 out of 5 mice and using a serum dilution of 1:2000 unless noted. Individual Western and ELISA results are in Supplementary Fig. 1a–q.

Biolistic immunization with the gene gun uses a burst of gas to propel DNA-bound gold particles to the dermal tissues (Fig. 2a). This method of genetic immunization leads to direct transfection of dendritic cells^28^ and *in vivo* expression of the encoded protein in both the dermal tissues and the lymph nodes^28^,^29^. Although biolistic immunization is more technically challenging than immunization by DNA needle injection, biolistic delivery effects a Th2 response, which is specifically associated with antibody production, in comparison to the Th1 response favored upon DNA injection^30^.

The DNA-gold particles used in this study were micronanoplexes (Fig. 2a)^31^. Micronanoplexes are complexes of two differently-sized gold particles: micron-sized (1–2 μm) gold particles that are coated with polyethylenimine (microgold-PEI; Fig. 2a)^32^, and nanometer-sized gold particles that are generated upon modification with cysteamine and DNA (DNA-nanogold; Fig. 2a)^31^,^33^. The micron-sized particles allow dermal penetration, and the nanometer-sized particles provide a high surface area for DNA binding. Micronanoplexes allow an order of magnitude higher DNA-binding capacity compared to micron-sized particles alone^31^. Micronanoplexes have previously been used to express luciferase in mice^31^ and to identify vaccine antigen candidates that provided protection against the bioterrorism agent *Burkholderia mallei*^34^. The results presented here represent the first described application of DNA-gold micronanoplexes to antibody production.

To generate polyclonal antibodies, each antigen-encoding gene was cloned as a full-length open reading frame with its natural codon usage into the novel vector pCMVi-LSrCOMPTT (Fig. 2b), and several targets were also cloned into the vaccine construct pCMVi-UB (Fig. 2c)^35^. pCMVi-LSrCOMPTT is based on vectors that were used to produce antibodies against proteins lacking transmembrane domains^15^. pCMVi-LSrCOMPTT allows expression of the target membrane protein as a fusion with a 174-residue sequence containing the following four immune-stimulating domains (Fig. 2b). “LS” is the 24 amino acid secretion leader sequence from the human α1-antitrypsin gene^36^, which allows targeting of the fused protein to the plasma membrane^37^. To our knowledge, this work is the first reported use of the LS sequence for generation of antibodies against membrane proteins. “r” is a randomly-generated 23 amino acid peptide that was previously shown to be immunogenic^15^. “COMP” is the 45 amino acid cartilage oligomeric matrix protein assembly domain from the pentameric rat matrix protein^38^. “TT” is a 50 amino acid sequence containing tetanus toxoid epitopes, which has been used to overcome humoral tolerance^39^. All plasmids used in this study and their sequences are available from the PSI:Biology-Materials Repository at DNASU^40^ (Supplementary Table 2).

A typical immunization schedule was applied and consisted of a double prime plus 2–4 genetic boosts until target-specific ELISA titers of 1:1000 were achieved, in comparison to naïve mice (Supplementary Fig. 1a–q). Antibody specificity was confirmed in Western blots (Fig. 2d and Supplementary Fig. 1a–q). Biolistic immunization using pCMVi-LSrCOMPTT yielded antibodies for 12 membrane proteins, including 11 of 14 targets from *F. tularensis* and one of three targets from ASFV (Fig. 2d). Using altered adjuvants, improved titers were obtained with fewer boosts (Supplementary Fig. 2a–k), which suggests the possibility of further enhancement upon systematic investigations.

For nine targets that yielded negative immunoblots at a serum dilution of ≤1:500, the corresponding genes were cloned into pCMVi-UB (Fig. 2c)^35^, which allows expression of the target as a fusion with mouse ubiquitin. pCMVi-UB has been used previously to evaluate protective antigens in vaccine studies (reviewed in^41^) but has also yielded strong antibody responses^35^,^42^. The ubiquitin sequence contains the Gly76Ala mutation to prevent de-ubiquitination^43^. The expressed protein is not deliberately membrane-directed and may be expected to undergo MHC I presentation. We found that pCMVi-UB yielded a high response for the p54 envelope protein from ASFV (Fig. 2d and Supplementary Fig. 3a,b). ELISA responses for the other eight targets were not appreciably higher than in the presence of negative control IVT-HMB products (Supplementary Fig. 3a, dashed lines). The lack of measurable response for these eight targets is not an unbiased predictor of antibody generation by pCMVi-UB, since the antigens tested also yielded moderate or no reactivity in pCMVi-LSrCOMPTT.

### Boosts with protein from IVT-HMB

We further noted that boosting with antigen from IVT-HMB reactions yielded higher ELISA titers (Supplementary Fig. 2f,j,k). However, this reactivity may be due in part to irrelevant proteins (Supplementary Fig. 3c), possibly those visible as background bands in gels containing captured antigen (Fig. 1b,c). It is also not apparent whether the captured antigen contains folded domains. Nevertheless, for the eight pCMVi-UB constructs that lacked a DNA response (Supplementary Fig. 3a), target-specific reactivity was not observed in immunoblots following two boosts with IVT-HMB protein.

### Method appraisal

Importantly, success with this genetic immunization approach generally correlated with previous reports of target-specific immunogenicity in infected and immunized hosts (Supplementary Table 1, column “Reported immunogenicity”). The exceptions were CD2v, for which host recognition has been described for only a highly glycosylated form^44^, and *F. tularensis* FupB, for which antibodies were raised in rats from a recombinant fragment^45^. The efficiency of our method (71%) also approaches that described for non-transmembrane proteins, which was 84% (n = 100) using biolistic immunization with micron-sized gold and codon optimization^15^, and 78% (n = 18 proteins) or 83% (n = 6), respectively, using tail or limb vein injection of DNA^46^.

Our approach is expected to have wide applicability, given that robust antibodies were generated for more than half of the targets from two disparate hosts and across a structurally diverse set of membrane proteins. The targets included ten α-helical, seven β-barrel, and two lipidated proteins (Supplementary Table 1). Of interest will be whether this method can be adapted to better recognize targets that are poorly immunogenic by any approach, such as for ion channels that consist mainly of membrane-buried α-helices^47^ and for highly conserved targets whose autoreactive B cells would likely be removed by tolerance checkpoints^48^. Indeed, our approach yielded no measurable serum reactivity for the two most hydrophobic targets, FTT0759 and the CapC subunit of the capsule biosynthesis complex. This result is consistent with the well-known poor immunogenicity of hydrophobic regions, and is usually attributed to the low structural complexity of these stretches. It will be of interest to determine whether improved recognition can be achieved by the use of codon-optimized genes or by co-immunization of subunits that normally exist as part of a membrane protein complex (*F. tularensis* CapBCA^49^). Further method development may be guided by improved understanding of the membrane protein expression pathways undertaken by these targets upon immunization as well as the mechanisms of immune recognition upon biolistic immunization, which have generally focused on cellular, not humoral, immune responses and have not been specifically explored for integral membrane proteins.

### Antibody-based characterization of membrane-targeting upon recombinant expression

To support structural studies of membrane proteins involved in pathogenesis, we used the antibodies to characterize membrane-targeting of several proteins from *F. tularensis* upon recombinant expression in *Escherichia coli*. Because several targets could not be purified when expressed as fusions with purification tags, we analyzed expression of targets that lacked purification tags. This tag-free approach evaluated whether membrane-targeting alone could be achieved in *E. coli* using the native *F. tularensis* protein sequences and independently of other *F. tularensis*-specific factors.

**Figure 3:**
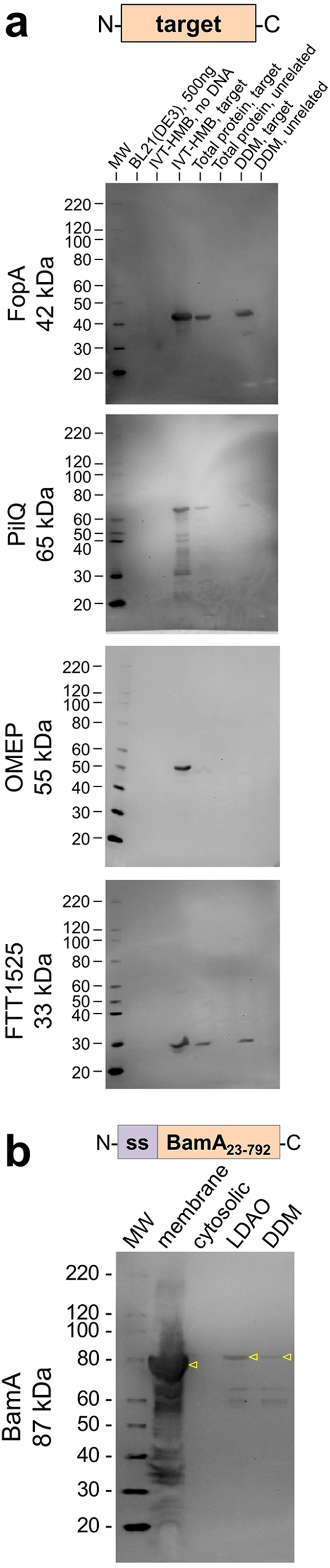
Western analyses of tag-free *F. tularensis* membrane proteins identifies membrane-targeting upon recombinant expression. (**a**) Immunoblots probed with 1:2000 dilution of serum. IVT-HMB samples contain 100–200 ng of target protein, or beads from an equivalent reaction volume lacking template DNA. Total protein and detergent-solubilized samples contain protein from 16 μL of culture in which the target protein or an unrelated target was expressed. DDM, *n*-dodecyl β-D-maltoside. The unrelated targets used in the blots were, from top to bottom: PilQ, OMEP, FTT1525, and TolC. (**b**) Western analyses of the membrane fraction, cytosolic fraction, and detergent-solubilized proteins of the membrane fraction upon expression of *F. tularensis* BamA containing the signal sequence (ss) from *E. coli* BamA. The BamA protein is identified (yellow triangles). Lanes contain protein from the equivalent of 100 μL of culture. Serum dilution was 1:500. LDAO, *N*,*N*-dimethyldodecylamine *N*-oxide.

Western analyses using antibodies against four tag-less targets confirmed their expression *in vitro* (Fig. 3a, lane “IVT-HMB, target”). In contrast, *in vivo* expression in *E. coli* was undetectable for the outer membrane efflux protein (OMEP; Fig. 3a, lane “Total protein, target”). PilQ was poorly expressed *in vivo* (Fig. 3a, lane “Total protein, target”), and little of this membrane protein appeared to be membrane-directed as indicated by detergent solubility (Fig. 3a, lane “DDM, target”; DDM = *n*-dodecyl β-D-maltoside). These results suggest that OMEP and PilQ may require alternative strategies to generate these proteins for structural analyses. In comparison, *in vivo* expression was more apparent for the virulence determinants FopA and FTT1525 (Fig. 3a, lanes “Total protein, target”). Importantly, the antibody reagents generated in this study allowed membrane-targeting of FopA and FTT1525 to be demonstrated for the first time, by detection of these proteins in DDM-solubilized fractions (Fig. 3a, lanes “DDM, target”). These immunoblots additionally demonstrate the target specificity of some of the non-enriched sera obtained in this study, as evidenced by the lack of additional bands that represent proteins from host cells (Fig. 3a, lane “BL21(DE3)”) or proteins from IVT-HMB reactions (Fig. 3a, lane “IVT-HMB, no DNA”). Use of the remaining sera in future studies will require the application of appropriate controls^50^ to avoid misinterpretation of data.

Similar analyses with purification fractions of a recombinant form of the BamA major subunit of the *F. tularensis* β-barrel assembly machine indicated virtually no membrane-targeting in *E. coli* (Fig. 3b, lanes “LDAO” and “DDM”; LDAO = *N*,*N*-dimethyldodecylamine *N*-oxide). Evidence of strong expression (Fig. 3b, lane “membrane”) suggests that recombinant BamA may be a candidate for refolding from inclusion bodies, towards structural investigations.

## Conclusions

In summary, we used an immunization approach that precludes the need for purified protein^14^ to generate polyclonal antibodies against membrane proteins. To facilitate characterization of the antibodies, we developed a simple, modified *in vitro* reaction to simultaneously express and capture membrane protein antigen. Finally, Western analyses using these antibodies demonstrated antibody specificity and also identified promising candidate target membrane proteins for future structural studies. As studies with these membrane proteins progress, monoclonal antibodies will be generated from B cells isolated from genetically immunized hosts (Supplementary Methods). The techniques and materials described here will support the elucidation of the mechanisms of pathogenesis and the development of infectious disease therapies.

## Methods

### Membrane protein targets

Membrane proteins in this study (Supplementary Table 1) were selected by the Center for Membrane Proteins in Infectious Diseases (MPID; grant GM094599), as part of the PSI:Biology program under the U.S. National Institutes of Health’s Protein Structure Initiative. Information on MPID targets is available at the Structural Biology Knowledgebase Technology Portal^51^ (http://sbkb.org). Due to technical difficulties that are commonly encountered when working with membrane proteins, the methods are presented here in necessary detail.

### General cloning information

Genes encoding the membrane proteins were amplified from genomic DNA using iProof High-Fidelity DNA Polymerase (Bio-Rad #172-5302). *Francisella tularensis* subsp. *tularensis* SCHU S4^21^ genomic DNA was provided by Drs. C. Rick Lyons, Terry H. Wu, and Jason Zsemlye (University of New Mexico). ASFV genomic DNA from the highly virulent isolates Georgia 2007/1^52^ and Malawi Lil 20/1^53^ was provided by Dr. Linda K. Dixon (The Pirbright Institute, United Kingdom). Unless noted, cloning was achieved using the In-Fusion HD Cloning Plus system (Clontech #638910). This ligation-independent system allows simple matching between 15 base pairs of each end of a PCR-derived insert with the ends of the linearized parent vector. Plasmid DNA was prepared with the QIAprep Spin Miniprep or Plasmid Maxi system (QIAGEN #27106 and #12163, respectively). DNA elutions were done using molecular grade water. Sequence confirmation of the complete insert in each plasmid clone was performed at the School of Life Sciences DNA Laboratory at Arizona State University. Vectors used in this study and representative primers are in Supplementary Tables 2 and 3, respectively.

### Vectors for *in vitro* protein expression

Protein used to characterize most of the sera was generated in IVT-HMB reactions from pRSET-natGFP constructs (Fig. 1b), which yields target protein fused to the C-terminal folding reporter GFP sequence^54^. Ligation-independent cloning into pRSET-natGFP and pRSET-natGFPHis was accomplished using *Bse*RI-digested vector. Because cleavage occurs outside of the *Bse*RI recognition site, and owing to vector design^55^, digestion with *Bse*RI in these vectors circumvents inclusion of any *Bse*RI recognition site sequence in the resulting subclones.

Membrane proteins that were used in protein boosts and for some sera analyses were prepared from pET-32b-TEV constructs (Supplementary Fig. 4). pET-32b-TEV allows expression of the target membrane protein as a fusion with an N-terminal sequence consisting of *E. coli* thioredoxin, hexahistidine, thrombin cleavage site, S-tag, and TEV protease cleavage site (Supplementary Fig. 4). Target sequences with stop codons were cloned in-frame into pET-32b-TEV using the *Bam*HI and *Hin*dIII restriction sites, except that ASFV p54 used *Bam*HI and *Sal*I.

### Linear expression element (LEE) constructs for *in vitro* protein expression

Full-length forms of FTT0759 and CD2v could not be obtained from IVT-HMB reactions as GFP-fusions using pRSET-natGFP or as thioredoxin-fusions using pET-32b-TEV. Therefore, these two targets were expressed as multiple target fragments from PCR-generated linear expression elements (LEEs)^56^ (Fig. 1c). The constructed LEEs generated each target fragment as a fusion with N-terminal thioredoxin and C-terminal histidine-tag sequences (Supplementary Fig. 5a–f). Schematics of the LEEs for FTT0759 and CD2v and are shown in Supplementary Fig. 5a,d, respectively. The expressed ORF is flanked by a transcription promoter, translational signals, and a transcription terminator (Supplementary Fig. 5a,d). Detailed DNA and protein sequences of representative LEEs for FTT0759 and CD2v are in Supplementary Fig. 5b,c,e,f. Oligonucleotides used for generation of LEEs are detailed in Supplementary Table 4.

FTT0759 fragments were selected to avoid interruption of predicted transmembrane helices. Alpha-helical transmembrane segments were predicted using TMHMM^57^, SOSUI^58^, TopPred^59^, and TMPred^60^. The following fragments for FTT0759 were obtained by IVT-HMB: amino acid residues 1–158, 149–250, 59–128, 59–192, and 120–220 (Fig. 1c). IVT-HMB products were not obtained for the following fragments: 59–220, 59–305, 149–305 and 179–305. Therefore, no products were obtained for all three fragments encompassing residues 251–305.

LEE template DNA for use in IVT-HMB reactions was generated in three steps. (1) Each of the three LEE DNA segments shown at the bottom of Supplementary Fig. 5a,d was generated in a 30 μL reaction using Bio-Rad iProof High-Fidelity DNA Polymerase, 250 μM of each dNTP (Life Technologies #10297-018), 5–10 ng of template DNA, and 200 μM of each primer. Reaction conditions were 98 °C × 30 s; 27–30 cycles of (98 °C × 10 s; 55 °C × 30 s; 72 °C × 15 s/kb); and 72 °C × 1 min. Products were gel-purified with a QIAquick Gel Extraction Kit (Qiagen #28706) and quantified by absorbance at 260 nm. (2) LEE segments were assembled into a full-length product in a 30 μL reaction using iProof High-Fidelity DNA Polymerase, 250 μM of each dNTP, and 0.16 pmol of each LEE segment from step (1). Reaction conditions were 98 °C × 30 s; 10 cycles of (98 °C × 10 s; 55 °C × 30 s; 72 °C × 1 min/kb); and 72 °C × 10 min. (3) Full-length LEEs were amplified in a 50 μL reaction using PrimeSTAR Max DNA Polymerase (Clontech #R045), 2 μL of the assembly reaction from step (2), and 10 μM of each primer T7Pro-FOR and T7Ter-REV. Reaction conditions were 30 cycles of (98 °C × 10 s; 55 °C × 15 s; 72 °C × 10 s/kb). A 2 μL sample was analyzed on an agarose gel. DNA from the remaining reaction was purified using the Agencourt AMPure XP PCR magnetic particle purification system (Beckman Coulter #A63880), eluting with 40 μL of water. This AMPure purification step was critical for obtaining high protein yields in IVT-HMB reactions. DNA products were quantified by absorbance at 260 nm.

The 3′ LEE fragments in Supplementary Fig. 5a,d were originally constructed from multiple PCR reactions. For future use as template DNA, one of each LEE for FTT0759 and CD2v was subcloned into the pJET1.2 vector using CloneJET PCR Cloning (Clontech #K1231) and sequence-verified, yielding subclones pJET1.2-LEE-Trx-FTT0759aa59–128 and pJET1.2-LEE-Trx-EP402Raa10–202.

### Vectors for genetic immunization

pCMVi vector constructs are designed to express target proteins upon genetic immunization of mice^15^. The pCMVi vectors described in this study contain the cytomegalovirus (CMV) immediate-early promoter enhanced by a chimeric intron (human β-globin and an immunoglobulin gene) upstream of the target gene, and the human growth hormone terminator downstream of the target gene.

The novel vector pCMVi-LSrCOMPTT (Fig. 2b) contains the backbone of pCMVi10^15^ plus three alterations: (i) the tetanus toxoid epitopes from pBQAP-TT^15^ were added between the COMP domain and the target ORF, (ii) the transcription terminator from the human growth hormone gene replaced that from the rabbit β-globin gene^14^, and (iii) a strong Kozak sequence^61^ was included near the “LS” secretion leader sequence. Target ORFs with stop codons were cloned in-frame into pCMVi-LSrCOMPTT using the *Bgl*II and *Hin*dIII sites, except ASFV p54 used *Bgl*II and *Xba*I. CD2v was also cloned into the pCMVi-LS vector^35^.

Nine target ORFs were cloned into pCMVi-UB^35^ in-frame with and 3′ of the mouse ubiquitin sequence using the *Bgl*II and *Bam*HI restriction sites. The CD2v ORF in pCMVi-UB was derived from protein expression constructs and therefore had been optimized in the first nine codons for expression in *E. coli*.

### Adjuvant vectors for genetic immunization

pCMVi-LS-LTA-R192G and pCMVi-LS-LTB encode the enterotoxigenic *E. coli* heat-labile enterotoxin subunits A (mutant) and B, respectively^34^. An amendment of the cloning method is described here. For both plasmids, the large *Eco*RI/*Xba*I fragment of pCMVi-UB^35^ provided the backbone, into which the following sequence was generated using overlapping oligonucleotides: a Kozak sequence, the N-terminal 24 amino acid leader sequence (LS) from the human α1-antitrypsin gene, and a mouse-codon-optimized sequence encoding the enterotoxigenic *E. coli* heat-labile enterotoxin subunit A (LTA; amino acids 30–269 of NCBI accession no. ABV16233.1, with mutations R192G and N189D) or subunit B (LTB; amino acids 2–104 of NCBI accession no. ACJ23372.1). The R192G mutation eliminates toxicity by abolishing proteolytic processing of the A subunit, while still conferring adjuvant activity^62^.

### CpG oligodeoxynucleotide adjuvants

To stimulate antibody production, unmethylated CpG oligodeoxynucleotides (ODNs) that mimic the presence of foreign DNA (reviewed in^63^) were included as adjuvants. Class B CpG ODN 2006 (5′-TCGTCGTTTTGTCGTTTTGTCGTT-3′) with complete phosphorothioate linkages was purchased from Sigma. Class C CpG ODN 2395 (5′-TCGTCGTTTTCGGCGCGCGCCG-3′) with complete phosphorothioate linkages was purchased from Life Technologies. CpG ODNs were suspended in water.

### Vectors for *in vivo* expression in *E. coli*

Tag-free expression constructs for FopA, PilQ, OMEP, and FTT1525 (Fig. 3a) were generated by cloning the ORFs with a strong double stop codon signal (TAATAA) into *Bse*RI-digested pRSET-natGFPHis^55^.

pCDF-BAD was generated by D.T.H. in the laboratory of Jeffrey L. Hansen (Medical University of South Carolina) by ligase cloning into the *Pac*I and *Xba*I sites of pCDFDuet-1 (Novagen), which confers spectinomycin-resistance and contains the CloDF13 origin of replication. The cloned insert contained the arabinose-inducible promoter and the *E. coli rrnB* T1T2 transcription terminator sequences from pBAD18^64^ separated by a novel polylinker/RBS sequence consisting of *Not*I-*Pme*I-AGAAGGAGATTA-*Nde*I-*Xho*I-*Asc*I-*Aat*II. The subclone pCDF-BAD-EcBamA-FTT1573 was generated using the *Nde*I and *Xho*I sites of pCDF-BAD. The expressed protein contains a homologous *E. coli* BamA signal sequence (amino acids 1–21 from NCBI accession no. P0A940) fused to the full-length *F. tularensis* BamA (amino acids 24–792) following signal sequence cleavage as predicted by SignalP 4.0^65^.

### Production of membrane proteins serving as antigens by IVT-HMB

The IVT-HMB (*in vitro* translation in the presence of hydrophobic magnetic beads) reaction was based on the Expressway Maxi Cell-Free *E. coli* Expression System (Life Technologies #K9900-97). Each 100 μL of the system’s Total Reaction solution consisted of 50 μL of Starter Reaction solution and 50 μL of Feed Reaction solution. Reactions were performed in 1.5 mL Protein LoBind tubes (Eppendorf #022431081) or in 96-well plates (1.2 mL per square well, U-bottomed, with sealing lids; ABgene #AB-1127). Due to the need to maintain suspension of the beads, yields were reproducible when using 50–400 μL of Total Reaction volume per tube or well. For each 100 μL of Total Reaction solution, 25 μL of Dynabeads M-280 Tosylactivated (Life Technologies #142.04) were washed three times in phosphate buffered saline (PBS; 137 mM NaCl, 2.7 mM KCl, 10.1 mM Na_2_HPO_4_, 1.8 mM KH_2_PO_4_, pH 7.4) by pelleting on a magnet (Life Technologies #123-20D). The 50 μL Starter Reaction solution was prepared on ice by combining the following Expressway System components: 20 μL of *E. coli slyD*^*−*^ extract; 20 μL of 2.5X IVPS Rxn Buffer; 1.25 μL of 50 mM amino acids solution (19 amino acids, without methionine); and 1 μL of 75 mM methionine. Subsequently added was 1 μL of a solution prepared at room temperature and consisting of one tablet of Complete Mini, EDTA-free Protease Inhibitor (Roche #11836170001) dissolved in 200 μL of room-temperature water; 1.25 μL of RNase Inhibitor at 40 units/μL (New England BioLabs #M0314L); and 1 μL of T7 Enzyme Mix from the Expressway System. The resulting 45.5 μL mixture was used to suspend the washed and pelleted beads. This bead mixture was added to a 4.5 μL solution containing the DNA template at the optimal concentration of 1 μg of plasmid DNA or 0.5 μg of AMPure-purified LEE DNA. When necessary, template DNA had been concentrated by speed vacuum centrifugation and then suspended in water. IVT-HMB reactions were incubated at 30 °C with shaking at 530–550 rpm, such as in a Thermomixer R (Eppendorf #022670107) or a HiGro Microplate Cell Incubator for 96-Well Plates (Digilab #JHGA0296OH). After 30 min, 50 μL of Feed Reaction solution was added, which consisted of 20.5 μL of water, 25 μL of Expressway 2X IVPS Feed Buffer, 1.25 μL of 50 mM amino acids solution (without methionine), 1 μL of 75 mM methionine, 1 μL of Protease Inhibitor solution, and 1.25 μL of RNase Inhibitor. Incubation was continued at 30 °C with shaking for 3.5 h. Beads were washed three times in PBS (200 μL per 100 μL of Total Reaction solution) before suspension in PBS and storage at −20 °C.

SDS-PAGE analyses of IVT-HMB products were done by suspension of magnet-pelleted beads in 1X SDS-PAGE Loading Solution, which was 1X XT Sample Buffer (Bio-Rad #161–0791) containing 715 mM of 2-mercaptoethanol. Prior to loading, these samples were heated at 90 °C for 5 min. Gels were 4–12% Bis-Tris polyacrylamide (Life Technologies #NP0329BOX) that were run in 50 mM 3-(*N*-morpholino)propanesulfonic acid, 50 mM tris(hydroxymethyl)aminomethane, pH 7.7, 0.1% sodium dodecyl sulfate (SDS), and 1 mM ethylenediaminetetraacetic acid (MOPS SDS Running Buffer, Life Technologies #NP0001). Visualization of the target proteins in SDS-PAGE (Fig. 1b,c) indicated that targets bind to the beads by hydrophobic interaction. In Fig. 1b,c, the lanes labeled “No DNA” indicate that template DNA was omitted in these IVT-HMB reactions. IVT-HMB protein yields were estimated by comparison to bovine serum albumin (BSA) standards (Bio-Rad #500-0007) run on the same gel following visualization by silver stain (Pierce #24612). Molecular weight standards were Precision Plus Protein Dual Color Standards (Bio-Rad #161-0374).

### Bullets for genetic immunization

The Helios gene gun (Bio-Rad #165-2451) uses a burst of helium gas to propel DNA-bound gold particles, which have been adhered on the inside of a piece of tubing, to the dermal tissues. Formation of DNA-gold micronanoplexes was based on Svarovsky *et al.*^31^ with slight modifications. Unless noted, molecular grade water and room temperature were used throughout this process. Bullets for the gene gun consisted of 1 cm length pieces of tubing that were coated on the inner surface with the DNA-gold micronanoplexes. Tefzel tubing (0.125 in. OD, 0.093 in. ID; Bio-Rad #165–2441) was washed with ethanol and then acetone by pulling the solutions through using a vacuum, followed by drying for 30 min by flowing inert gas through the tubing at a flowrate of 5 L/min. The washed tubing was stored in enclosed circles by connecting the ends with adapter tubing (silicone, 3/32 in. I.D., 5/32 in. O.D., Tygon 3350; Saint-Gobain #ABW00004). On the day of bullet preparation, the Tefzel tubing was cut into 33 cm lengths and attached to a non-Luer Lok 10 mL syringe, with plunger, via adapter tubing cut at a 1 cm length. The syringe barrel was retracted by approximately 1 mL to facilitate later expulsion of liquid.

The micron-sized particles (microgold-PEI) and nanometer-sized particles (DNA-nanogold) are prepared separately and then mixed to allow association between the positively-charged polyethylenimine and the negatively-charged DNA (Fig. 2a).

### Preparation of microgold-PEI

Micron-sized gold particles (microgold-PEI) (Fig. 2a) that were coated sequentially with 11-mercaptoundecanoic acid and polyethylenimine (PEI) were prepared as follows. Two grams of unmodified micron gold particles (Ferro, Inc. #J5G2000; or Bio-Rad #165–2264) were introduced to a glass flask. Within a fume hood, 15 mL of concentrated (98%) sulfuric acid was added. While mixing, 5 mL of 30% hydrogen peroxide was added dropwise to minimize exothermic heating. The solution was allowed to cool to room temperature for approximately 20 min. After decanting and discarding the supernatant, the gold was washed three times with 50 mL of water, transferred to a 50 mL polypropylene tube, and washed four times with 40 mL of water with mixing by vortex. The supernatant was decanted and discarded. The gold was washed twice with 20 mL of 100% ethanol. Centrifugation at 2000 rpm facilitated discarding of the supernatant. The gold was mixed with 20 mL of ethanol using a vortex, and 0.5 g of 11-mercaptoundecanoic acid (Sigma-Aldrich #450561; stored under desiccant) was added to allow alkanethiol modification of the gold surface. The solution was shaken horizontally at 1400 rpm for 2 h under protection from light and then centrifuged at 900 *g* for 2 min. After discarding the clear supernatant using a pipet, the gold was mixed by vortex in 20 mL of a 0.1 M solution of 2-(*N*-morpholino)ethanesulfonic acid (MES; brought to pH 6.0 with sodium hydroxide and 0.2 μm-filtered). Mixing by vortex followed addition of 300 mg of *N*-hydroxysuccinimide (Sigma-Aldrich #130672; stored under nitrogen and desiccant) and addition of 200 mg of *N*-(3-dimethylaminopropyl)-*N′*-ethylcarbodiimide hydrochloride (Sigma-Aldrich #E1769; purchased within 6 months and stored under nitrogen and desiccant), which activated the carboxylic acid groups of 11-mercaptoundecanoic acid for later amide bond linkage^66^ to PEI. The solution was shaken horizontally at 1400 rpm for 30 min in the dark. During this incubation, 2 g of branched PEI (average MW ~25,000; Sigma-Aldrich #408727) was vigorously vortexed in 15 mL of water in a polypropylene tube, and the pH was adjusted to 9 with ~1 mL of HCl in order to protonate the amine groups, before adjusting the final volume to 20 mL using water. After centrifugation of the gold slurry for 2 min at 900 *g*, the supernatant was decanted and discarded. The PEI solution was added to the gold pellet, mixed by vortex, and then shaken horizontally at 1400 rpm for 2 h in the dark. After centrifugation for 2 min at 900*g*, the supernatant was discarded via pipet. The pellet was washed twice with 40 mL of water and the supernatant discarded via pipet. The gold was frozen at −80 °C for 30 min prior to lyophilization overnight in the dark. The microgold-PEI was stored at 4 °C in the dark and under nitrogen, and used within 3 months, due to gradual loss of binding capacity. Binding capacity of the microgold-PEI can be verified by comparison of the absorbance at 260 nm of the supernatants from 10 μg of DNA incubated for 30 min in 0.1 mL of 0.1 M MES, pH 6.0, in the presence vs. the absence of 3 mg of microgold-PEI.

### Preparation of nanogold

Nanometer-sized gold particles modified with cysteamine (nanogold) (Fig. 2a) were prepared as follows. Upon receipt, a 1 gram stock of hydrogen tetrachloroaurate(III) trihydrate (Alfa Aesar #36400-03; or Sigma-Aldrich #520918-1G) was suspended in 5 mL of water to yield 508 mM of HAuCl_4_. This solution was stored at 4 °C under nitrogen gas and used within 6 months. Upon the day of bullet preparation, a 1.59 mM solution of HAuCl_4_ was prepared by adding 62.5 μL of 508 mM HAuCl_4_ to 20 mL of water. Under vigorous stirring and protection from light, 200 μL of 213 mM cysteamine (CAS number 60-23-1; Sigma-Aldrich #30070; or Santa Cruz #sc-217991) was added dropwise, upon which the yellow solution turned darker. Due to rapid oxidation of cysteamine, the cysteamine solution was freshly prepared from a recently-ordered, unopened bottle that had been maintained under nitrogen gas and desiccant. After 10 min of continuous stirring of the HAuCl_4_/cysteamine solution, 5 μL of a 10 mM solution of the reducing agent sodium borohydride (Sigma-Aldrich #480886; stored under desiccant) was rapidly injected directly into the stirring solution. The sodium borohydride solution had been prepared immediately before use and discarded immediately after use due to release of hydrogen gas upon its dissolution in water. Mixing of the nanogold suspension (1.59 mM HAuCl_4_, 2.13 mM cysteamine, 2.5 μM NaBH_4_) was continued for 30 min, upon which the color of the clear solution turned a deep wine red, indicating formation of gold particles of ~36 nm size^31^,^33^. The nanogold suspension was stored at 4 °C in the dark and under nitrogen, and used within 48 h.

### Preparation of DNA-nanogold

Cysteamine-modified nanogold was complexed with DNA to form DNA-nanogold (Fig. 2a) as follows. Plasmid DNA and CpG oligodeoxynucleotide solutions were combined in a polypropylene tube. Nanogold suspension (1.59 mM HAuCl_4_, 2.13 mM cysteamine, 2.5 μM NaBH_4_) was added such that, per bullet, 57 μL of nanogold suspension was mixed with ≤10 μg total of plasmid DNA plus CpG that was in a volume of ≤15 μL. If necessary to reduce the volume, plasmid DNA had been precipitated with ethanol and sodium acetate prior to suspension in water. The DNA-nanogold suspension was incubated at room temperature for 5 min, upon which the suspension turned from red to violet, indicating formation of complexes of 300–400 nm size^31^.

### Preparation of DNA-gold micronanoplexes and bullets

DNA-gold micronanoplexes were formed by combining microgold-PEI and DNA-nanogold (Fig. 2a) as follows. A 167 mg/mL slurry of microgold-PEI was prepared by combining, per bullet, 1 mg of lyophilized microgold-PEI with 6 μL of 0.1 M MES, pH 6.0. DNA-gold micronanoplexes were formed upon combining equivalent bullet-volumes of the microgold-PEI and DNA-nanogold solutions. The micronanoplexes mixture was incubated at room temperature with occasional mixing by vortex for 10 min, during which time the supernatant became less colored. This supernatant can be analyzed by ethidium-bromide-stained agarose gel electrophoresis in order to estimate DNA complexation into micronanoplexes. The gold was ethanol-washed three times by centrifugation at 100*g* for 1 min, removing and discarding the supernatant via aspiration, and vortexing upon addition of ethanol (0.1 mL per 1 bullet). These washes were also facilitated by gentle, repeated pipetting. The final supernatant was removed by aspiration and discarded.

Bullets were prepared in a fume hood. The ethanol-washed DNA-gold micronanoplexes were suspended in 55 μL (per bullet) of 1-butanol (Sigma-Aldrich #360465) via tube inversion and gentle pipetting. Immediately upon vortexing, the suspension was rapidly drawn into the horizontally-laid 33 cm-length Tefzel tubing via syringe aspiration, up to a maximum distance of approximately 24 cm into the tubing. The tubing was allowed to rest for 5–10 min to allow the gold to settle on the inner surface of the tubing. The butanol, now colorless, was gently expelled from the open end of the tubing using the attached syringe. The gold in the tubing was dried for 10–20 min by flowing helium through the tubing at a rate of 5 L/min, upon which the gold turned a lighter shade. Over-drying can inhibit release of the DNA-gold micronanoplexes upon firing. Release of the gold can be visualized by firing bullets against a piece of paper. The tubing was enclosed by circularization via adapter tubing until the time that the tubing could be cut into 0.5-inch lengths (Bio-Rad Tubing Cutter #165–2422), yielding approximately 19 bullets for each 24 cm length of gold-bound tubing. Bullets were stored in tubes containing desiccant (Drierite #21001) and used within 3 days.

### Genetic immunization of mice

All procedures were performed in accordance with protocols approved by the Institutional Animal Care and Use Committee at Arizona State University. Six- to eight-week-old female A/J and BALB/c mice were purchased from The Jackson Laboratory and maintained in-house for at least 1 week before use. A/J mice (5 mice/target) were chosen based on previous experimental data showing strong antibody production upon biolistic immunization with micron-gold^42^, and BALB/c mice were used for immunization of ASFV targets in pCMVi-LSrCOMPTT. Mice were housed on a 12 h dark/light schedule in Thoren Maxi-Miser cages containing Irradiated Teklad Sani Chips (Harlan #7990) and Ancare Nestlets. Cages were maintained on ventilated racks and under pathogen-free conditions. Mice were provided food (Teklad Global 18% Protein Rodent Diet; Harlan #2918) and water *ad libitum*.

Groups of five mice were genetically immunized in the ear skin pinnae using the gene gun and a helium pressure of 350 psi. The presence of less than five mice in the results (Supplementary Figs 1–3) indicates loss of the mouse. Each genetic immunization prime or boost consisted of two bullets, with one bullet delivered to the pinnae of each ear. A double-prime consisted of two shots per mouse on day 1, followed by two shots per mouse on day 2 or 3. The shots on these days were done on alternate sides of the ear pinnae. Naïve mice were untreated.

Immunization for the 17 pCMVi-LSrCOMPTT constructs were as follows. DNA amounts are per bullet. All targets were immunized with 1 μg of pCMVi-LSrCOMPTT construct and 1 μg of class C CpG as a double-prime on days one and three, plus two boosts at two week intervals. Third and fourth boosts were done at ≥2 weeks for some targets, depending on the titer, and contained 1 μg of pCMVi-LSrCOMPTT construct, 21 ng of pCMVi-LS-LTA-R192G, 104 ng of pCMVi-LTB, and 1 μg of Class B CpG. A third genetic boost was done for BamA, TolC, FupB, CapA, CapB, FTT1406, p54, CD2v, and C-type lectin. A fourth boost was done for CapA, FTT1406, p54, CD2v, and C-type lectin. Sera analyses from this set are in Supplementary Fig. 1a–q.

A separate immunization of five targets (PilQ, OMEP, BamA, Flpp3 and FTT1406) in pCMVi-LSrCOMPTT suggested improved responses using altered adjuvants and also allowed analysis of protein boosts using antigen from IVT-HMB reactions (Supplementary Fig. 2a–k). The targets were immunized with 1 μg of pCMVi-LSrCOMPTT construct, 21 ng of pCMVi-LS-LTA-R192G, 104 ng of pCMVi-LTB, and 5 μg of Class B CpG as a double-prime on days one and two. The first boost was done after two weeks with the same DNA except that the CpG was dropped to 1 μg, since release of DNA-gold was inhibited upon firing with 5 μg of CpG per bullet. A second boost with the same DNA as the first boost was done after 5 weeks for OMEP, BamA and FTT1406. Serum analyses for this set are in Supplementary Fig. 2a–k. Comparison with the first immunization set containing all 17 pCMVi-LSrCOMPTT constructs indicated generally higher responses were obtained using the adjuvants in this second immunization set, as follows. Higher ELISA titers were achieved with fewer DNA boosts for PilQ (Supplementary Fig. 2a, one boost vs. Supplementary Fig. 1a, two boosts) and BamA (Supplementary Fig. 2d, one boost vs. Supplementary Fig. 1c, three boosts), and with the same number of boosts (two) for OMEP (Supplementary Fig. 2c vs. Supplementary Fig. 1b). Similar titers were obtained for Flpp3 using fewer boosts (Supplementary Fig. 2g, one boost vs. Supplementary Fig. 1i, two boosts).

Immunization for the nine pCMVi-UB constructs (FupA, FupB, FTT0759, CapA, CapB, CapC, p54, CD2v, and C-type lectin) were as follows. The targets were immunized with 1 μg of pCMVi-UB construct, 21 ng of pCMVi-LS-LTA-R192G, 104 ng of pCMVi-LTB, and 2.5 μg of Class B CpG as a double-prime on days one and three. For CD2v, the 1 μg of target-specific DNA was equally split between pCMVi-UB and pCMVi-LS constructs. For all targets, a genetic boost after 2 weeks contained 1 μg of pCMVi-UB construct, 21 ng pCMVi-LS-LTA-R192G, 104 ng pCMVi-LTB, and 1 μg class B CpG. For this boost, pCMVi-LSrCOMPTT constructs were used instead of pCMVi-UB for CapA, CapB, and FupA upon comparison with the previous immunization set of ELISA titers following the double primes. Sera analyses for this set are in Supplementary Fig. 3a,b.

### IVT-HMB protein for boosts

Membrane protein derived from IVT-HMB reactions was used in protein boosts for BamA immunizations in pCMVi-LSrCOMPTT (Supplementary Fig. 2f), for FTT1406 in pCMVi-LSrCOMPTT (Supplementary Fig. 2j,k), and for the nine targets in pCMVi-UB (immunoblot results for p54 are in Supplementary Fig. 3c). IVT-HMB protein used in immunizations was derived from pET-32b-TEV constructs (Supplementary Fig. 4), except FTT0759 antigen was from two LEEs encompassing amino acids 59–128 and 120–220 (Fig. 1c), and CD2v antigen was from two LEEs encompassing amino acids 10–202 and 170–355 (Fig. 1c). Mice were injected intraperitoneally with 0.1 mL volume of a 50% (vol/vol) solution of the adjuvant alum (Life Technologies #77161) containing 1 μg of class C CpG plus 1 μg of target protein bound to magnetic beads. The protein-bound beads had been washed four times in PBS with a final suspension in PBS. Prior to injection, the boost solution was mixed at 600 rpm at room temperature for 30 min. Protein immunizations in pCMVi-UB constructs included two boosts with IVT-HMB protein. Positive immunoblot results for p54 in pCMVi-UB are in Supplementary Fig. 3c. Immunoblots for the other eight targets yielded no measurable target recognition at the lowest serum dilutions tested (1:400-1:1000), and further indicated that, as with p54, injection of IVT-HMB protein contributed to recognition of several irrelevant proteins (serum dilutions of 1:10,000 and 1:100,000).

### Serum preparation

Sera samples were obtained by submandibular bleeding^67^. Final bleeds were done by cardiac puncture under anesthesia by tribromoethanol. Sera were prepared using 400–600 μL or 3.5 mL venous blood collection tubes with clot activator and gel for serum separation (Becton, Dickinson #365956 or #367981, respectively). Serum was brought to a final concentration of 50% glycerol by adding sterile 80% glycerol. These sera were stored at −20 °C until use. Serum dilutions were based on the 50% glycerol preparations.

### ELISAs

To ensure that positive immune responses were relevant to only the target membrane protein, protein antigens used to characterize mouse sera lacked protein sequence tags that were also present in the immunization steps. For enzyme-linked immunosorbent assays (ELISA), 100 ng of protein from IVT-HMB was coated in wells overnight at 4 °C using 100 μL per well in 0.03 M NaHCO_3_, 0.02 M Na_2_CO_3_, and 96-well flat-bottom MaxiSorp plates (Nunc #439454). Wells were washed three times in TBST (20 mM Tris, 136 mM NaCl, pH 7.4, 0.05% Tween-20) on a BioTek ELx405 platewasher. Manual washing using a magnet was necessary when yields of IVT-HMB protein were less than 5 ng of target protein per μL of IVT-HMB reaction. Blocking was done at 37 °C for 1 h in 200 μL/well of TBST containing 3% BSA (heat shock fraction, pH 7, ≥98%; Sigma #A7906-500 G) followed by washing as above. Wells were incubated at 37 °C for 1 h in 100 μL/well of serum diluted in TBST containing 3% BSA, followed by washing performed as described above. Wells were incubated at 37 °C for 1 h in 100 μL/well of secondary antibody (goat anti-mouse IgG [H+L], HRP conjugate; Life Technologies #626520) at a 1:3000 dilution in TBST containing 3% BSA, followed by washing as above. Detection was done by addition of 100 μL per well of ABTS (2,2′-azino-di[3-ethyl-benzthiazoline-6-sulfonate]) Peroxidase Substrate, 1-Component (KPL #50-66-06), incubation at 37 °C for 30 min, addition of 100 μL per well of 0.5 M HCl, and measurement at 415 nm in a Molecular Devices SpectraMax 190 plate reader.

### Western blots

Immunoblot analyses of polyclonal antibody containing sera used SDS-PAGE gels as described above. Transfer to pre-wet nitrocellulose (Pall #66485) was performed at 10 V and 4 °C overnight in chilled Tris-glycine (25 mM Tris, 192 mM glycine, pH 8.3) containing 20% methanol and using a Mini Trans-Blot Electrophoretic Transfer Cell (Bio-Rad #170-3930). Blocking and antibody steps were done on an orbital shaker at 0.5 revolution/s. Blocking was in TBST containing 5% BSA for 1 h at 37 °C or 2 h at room temperature. Subsequent steps were performed at room temperature. The membrane was incubated with primary antibody diluted in TBST containing 1% BSA for 1 h, then washed by rinsing twice in TBST and incubating twice for 5 min in TBST. The membrane was incubated with secondary antibody (goat anti-mouse IgG [H+L], HRP conjugate) at a 1:2000 dilution in TBST containing 1% BSA for 1 h, and then washed as above. Detection was with the SuperSignal West Pico Chemiluminescent Substrate (Thermo Scientific #34080) and using a Bio-Rad ChemiDoc XRS System. Molecular weight markers for immunoblots were the MagicMark XP Western Protein Standard (Life Technologies #LC5603).

### Protein expression and detergent-solubilization

Plasmid constructs encoding the four tagless forms of *F. tularensis* membrane proteins in Fig. 3a were expressed using fresh transformants of *E. coli* C43(DE3) as follows. Cultures were grown at 37 °C with shaking at 250 rpm in 14 mL snap-cap tubes (Corning #352057) containing LB medium (10 g peptone, 5 g yeast extract, 5 g NaCl) and 50 μg/mL carbenicillin. Pre-warmed medium (5 mL) was inoculated with 300 μL of overnight culture. When the culture absorbance at 600 nm reached 0.5–0.8, expression was induced with 0.5 mM of isopropyl β-D-1-thiogalactopyranoside. After 3 h, cell pellets were harvested from each 50 μL (for total protein) and 3 mL (for detergent-solubilization) of culture and stored at −20 °C.

Detergent-solubilized fractions of the four tagless *F. tularensis* membrane proteins were prepared from total cell protein obtained from 3 mL of culture as follows. All steps were done at room temperature unless noted. Cell pellets were thawed for 5 min prior to suspension in 250 μL of 50 mM sodium phosphate, pH 7.3, 300 mM NaCl, 10 mM imidazole, 1% DDM (>99.5%, for crystallography; Glycon #D97002-C), 2 mg/mL of chicken egg white lysozyme (Sigma #L6876), 0.01 mg/mL deoxyribonuclease I from bovine pancreas (EMD Millipore #260913), and 20 mM MgCl_2_, plus 1 tablet of Complete Mini, EDTA-free Protease Inhibitor per 10 mL of solution. The cell suspension was pipetted up & down ten times using a 1000 μL tip and then incubated for 15 min, in which every 5 min the samples were mixed by vortex for 5 s. To ensure lysis, three freeze-thaw cycles were done by incubating the sample at −80 °C for at least 10 min, thawing completely, and mixing by vortex. Samples were subjected to three cycles of 2 min of room-temperature bath sonication (Health-Sonics Corporation 0.8 Amp model #T1–9 C) plus 1 min on ice. Samples were centrifuged at 17,000 *g* for 15 min at 4 °C. The supernatant containing DDM-solubilized proteins was removed and stored at −20 °C. For Fig. 3a, 10 μL of the DDM-solubilized sample was combined with 5 μL of 4X SDS-PAGE Loading Solution, of which 2 μL was run on SDS-PAGE. For the total protein samples in Fig. 3a, the cell pellet from 50 μL of culture was suspended in 25 μL of 1X SDS-PAGE Loading Solution, of which 10 μL was loaded. Protein concentration of BL21(DE3) (Fig. 3a) was determined by Bradford assay (Bio-Rad #500-0002).

Membrane-targeting of *F. tularensis* BamA (Fig. 3b) was analyzed by detergent-solubilization of a membrane fraction as follows. *F. tularensis* BamA containing the signal sequence from *E. coli* BamA (EcBamA_1–20_-FTT1573_23–792_) was expressed in BL21(DE3) using construct pCDF-BAD-EcBam-FTT1573. Growth was at 37 °C in 1 liter of LB medium containing 50 μg/mL spectinomycin. When the absorbance at 600 nm reached 0.6, expression was induced with 0.2% arabinose. Cells were harvested after 3 h and frozen in liquid nitrogen prior to storage at −80 °C. The cell pellet (2.5 g, wet weight) was thawed, suspended in 10 mL of chilled Buffer A (50 mM HEPES, pH 7.3, 300 mM NaCl, and 1 Protease Inhibitor Cocktail Tablet, EDTA-Free [Sigma #S8830]), and sonicated for three 30 s cycles at 50% duty cycle and 50% power using an ultrasonic homogenizer Model 300 V/T (Biologics, Inc.). Additional Buffer A was added to bring the total volume to 26 mL prior to centrifugation at 100,000*g* for 40 min at 4 °C. The supernatant was removed. The pelleted membrane fraction (0.6 g) was suspended in 2.4 mL of Buffer A and homogenized using a Teflon-in-glass homogenizer (Corning #7724 T-5) at 4 °C. To 1.8 mL of the membrane suspension was added 225 μL of 10% (wt/vol) LDAO and 225 μL of glycerol. To 0.9 mL of the membrane suspension was added 100 μL of 10% (wt/vol) DDM. The membrane-detergent suspensions were gently shaken for 1 h at 4 °C prior to centrifugation at 16,800*g* for 40 min at 4 °C. Supernatant and pellet samples were frozen in liquid nitrogen and stored at −80 °C until the Western was performed as described above and using a 1:3000 dilution of secondary antibody.

## Additional Information

**Data Availability**: The sequences and DNA for plasmids reported in this study are deposited in the DNASU Plasmid Repository (https://dnasu.org/).

**How to cite this article**: Hansen, D. T. *et al.* Polyclonal Antibody Production for Membrane Proteins *via* Genetic Immunization. *Sci. Rep.* **6**, 21925; doi: 10.1038/srep21925 (2016).

## Supplementary Material

Supplementary Information
