# Supplementary material for: Polyclonal Antibody Production for Membrane Proteins via Genetic Immunization
Source: Sci Rep. 2016 Feb 24;6:21925. doi: 10.1038/srep21925 (PMC4764931; doi:10.1038/srep21925)
Supplement: Supplementary Information [file srep21925-s1.pdf]

Supplementary Information for

Polyclonal Antibody Production for Membrane Proteins *via* Genetic Immunization

Debra T. Hansen, Mark D. Robida, Felicia M. Craciunescu, Andrey V. Loskutov, Katerina Dörner, John-Charles Rodenberry, Xiao Wang, Tien L. Olson, Hetal Patel, Petra Fromme, Kathryn F. Sykes

**Contents**	**page**
Supplementary Methods	S2
Supplementary Figures 1 – 5	S3
Supplementary Tables 1 – 4	S25
Supplementary References	S32

SUPPLEMENTARY METHODS

Storage of splenocytes. Monoclonal antibody production does not require specialized procedures when combined with genetic immunization, however, the use of the A/J mouse strain to generate hybridomas is addressed here. The A/J strain has been used previously to generate monoclonal antibodies upon splenocyte fusion with a BALB/c-derived myeloma cell line and propagation in ascites within BALB/c mice or hybrid (BALB/c X A/J) CAF_1 mice[1-3]. For later monoclonal production, B cells were isolated from the mice described in Supplementary Figs. 2 and 3 and stored as follows, based on established protocols[4-6]. A freshly-isolated spleen was transferred to 15 mL of RPMI-1640 Medium (ATCC #30-2001). The following steps were done sterilely and at room temperature. The spleen was smashed in a small sterile petri dish with 5 mL of the medium and using the flat end of a plunger from a 10 mL syringe. Cells were passed through a cell strainer with 40 µm nylon mesh (BD #352340) that was set atop a 50 mL tube. The remaining medium was used to rinse the plunger and mesh. The cells were transferred to a 15 mL tube, debris was allowed to settle for 2 min, the supernatant was transferred back to the 50 mL tube, and the debris discarded. Cells were centrifuged at 800*g* for 5 min, and the supernatant was decanted and discarded. 10 mL of fresh medium was added, centrifugation was repeated, and the supernatant discarded. A 2 Ml volume of Red Blood Cell Lysing Buffer, Hybri-Max (Sigma #R7757), was used to completely suspend the cells by gentle pipetting, followed by incubation for 1 min, and then addition of 10 mL of medium. Cells were washed twice by centrifugation and suspension in 10 mL and then 5 mL of medium. Cells were passed through 40 µm mesh and maintained at 37 °C until use. Live cells counts used Trypan Blue Solution, 0.4% (w/v) in normal saline (CellGro #25-900-CI). Cells were adjusted to 10^7 cells/mL by centrifugation and suspension in 90% Fetal Bovine Serum, Heat Inactivated (Gibco #10438-026), and 10% tissue culture grade DMSO. 1 mL of cells was transferred to a 2 mL cryovial, which was placed in a room-temperature cryocooler and stored at -80 °C for 1-2 days until transfer to -150 °C.

SUPPLEMENTARY FIGURES

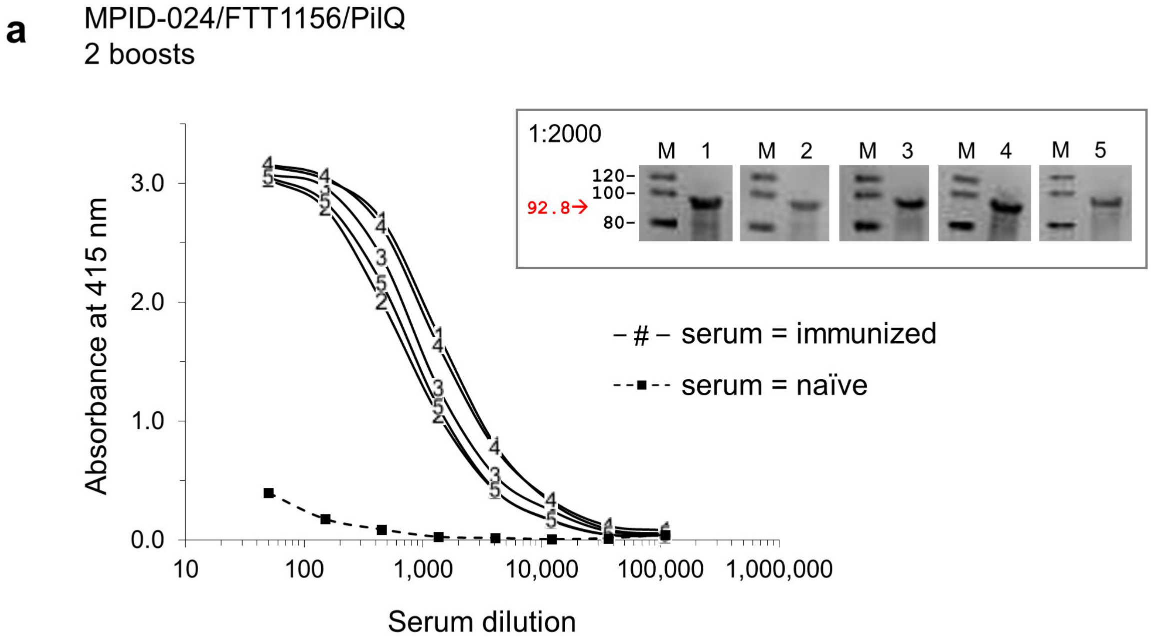

Supplementary Figure 1. Reactivity by ELISA and Western of sera from mice immunized with pCMVi-LSrCOMPTT constructs. Mice were immunized with pCMVi-LSrCOMPTT constructs that encode for 17 membrane proteins from *F. tularensis* and ASFV. (**a**) PilQ. (**b**) OMEP. (**c**) BamA. (**d**) TolC. (**e**) FopA. (**f**) FupA. (**g**) FupB. (**h**) FTT0759. (**i**) Flpp3 lipoprotein. (**j**) FTT1525. (**k**) CapA. (**l**) CapB. (**m**) CapC. (**n**) FTT1406. (**o**) p54 (Malawi). (**p**) CD2v. (**q**) C-type lectin. The total number of boosts by genetic immunization are listed. Immunization conditions are detailed in the Methods. Antigen with a C-terminal GFP was used in ELISAs and immunoblots and was generated by IVT-HMB using pRSET-natGFP constructs. Exceptions were the two partial-length targets (Fig. 1c): FTT0759 antigen fragments were derived from linear expression elements (LEEs)[7] encompassing amino acids 1-158 and 149-250, and CD2v antigen fragments were from LEEs encompassing amino acids 10-202 and 170-355. The FTT0759 and CD2v LEEs generated target protein fusions containing thioredoxin and a His-tag (diagrams of these constructs are in Supplementary Fig. 5a,d). The numbers in the ELISA graphs and above the immunoblots indicate the corresponding mouse number. Reactivity was determined for individual mice or for a pool containing an equal volume of serum from each of the available mice. “Naïve” indicates serum from one untreated mouse. For the ELISAs, each well contained 100 ng of target protein. Antigen from mock IVT-HMB reactions was generated in the absence of template DNA. For immunoblots, each lane contained 125 ng of target protein. Lanes containing molecular weight marker are indicated (M). Red text indicates the expected molecular weight of the IVT-HMB membrane protein product; migration outside of the expected position is typical of membrane proteins due to partial denaturation by detergents[8,9]. The serum dilution used in the immunoblots is listed in the immunoblot inset. Not shown are negative immunoblots, which were defined as blots that fulfilled any of the following criteria: (i) absence of a band within 20 kDa of the expected molecular weight; (ii) presence of the same apparent band in a lane containing 0.5 µg of total protein from *E. coli* BL21(DE3) that was not transformed with the target ORF; or (iii) presence of the same apparent band in a lane containing product from a comparable volume of an IVT-HMB reaction that lacked template DNA.

Supplementary Figure 1 (continued)

b MPID-025/FTT1258/OMEP
2 boosts

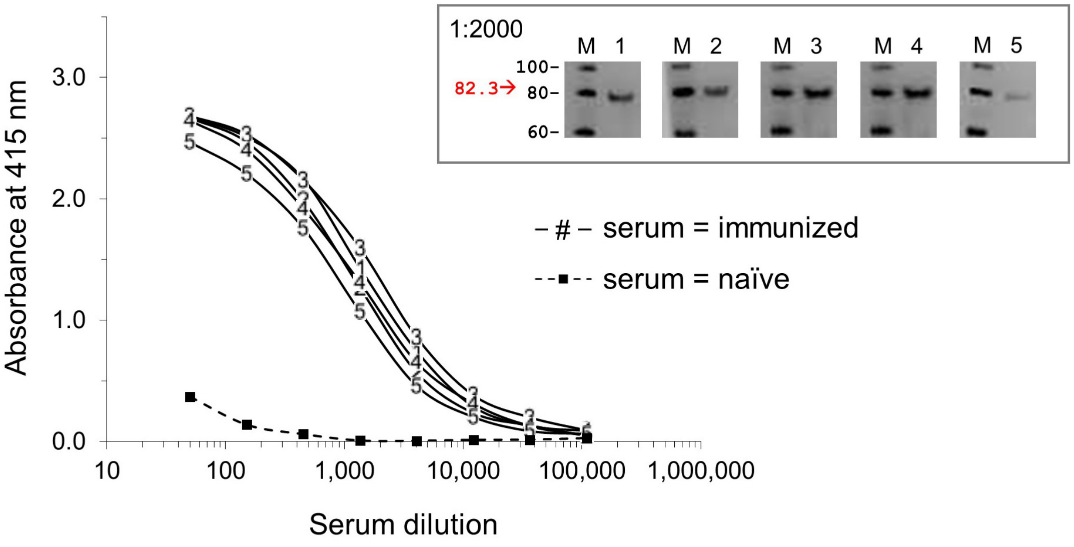

c MPID-026/FTT1573/BamA
3 boosts

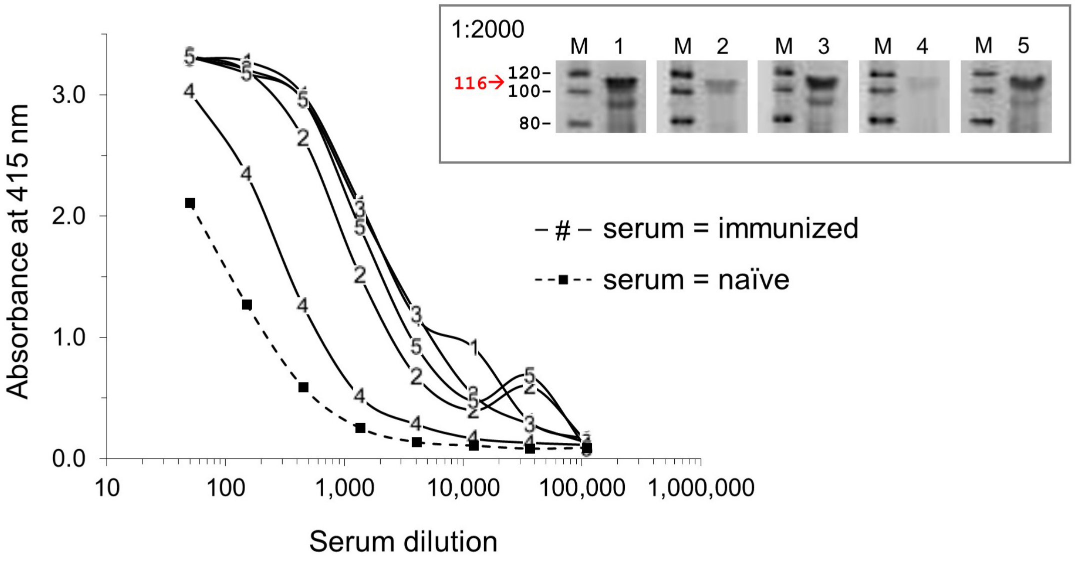

Supplementary Figure 1 (continued)

d MPID-027/FTT1724/TolC
3 boosts

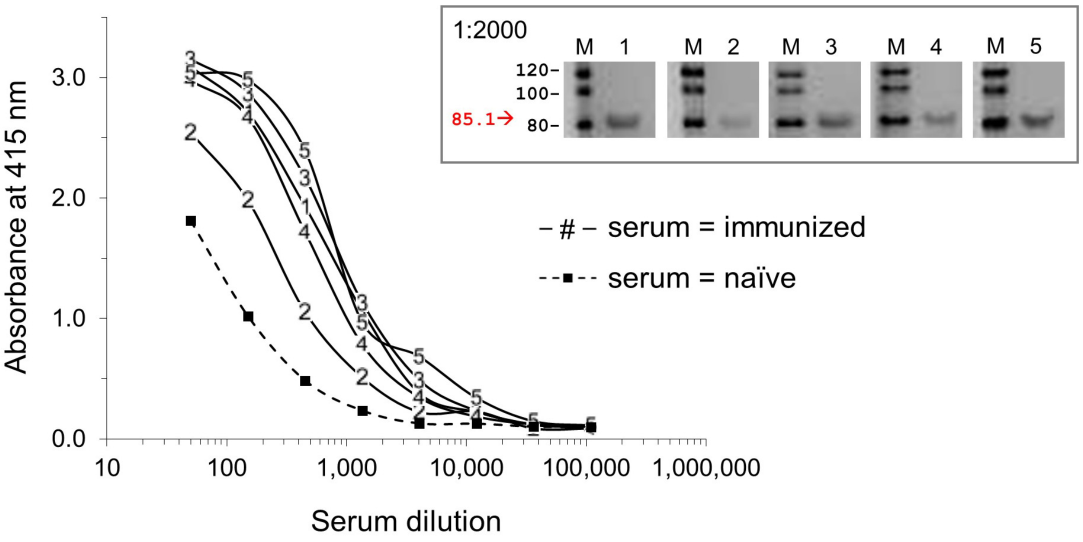

e MPID-028/FTT0583/FopA
2 boosts

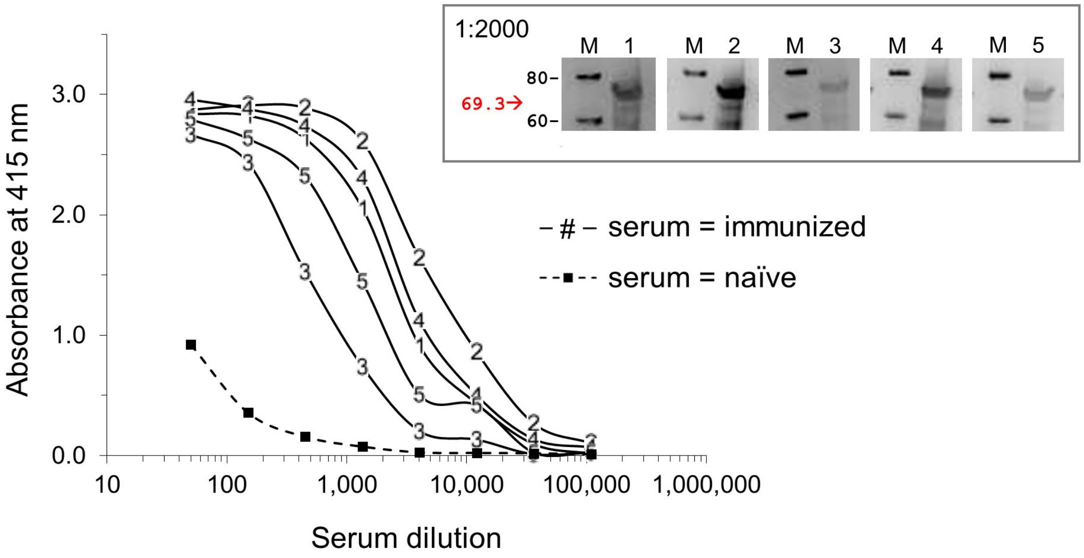

Supplementary Figure 1 (continued)

f MPID-029/FTT0918/FupA
2 boosts

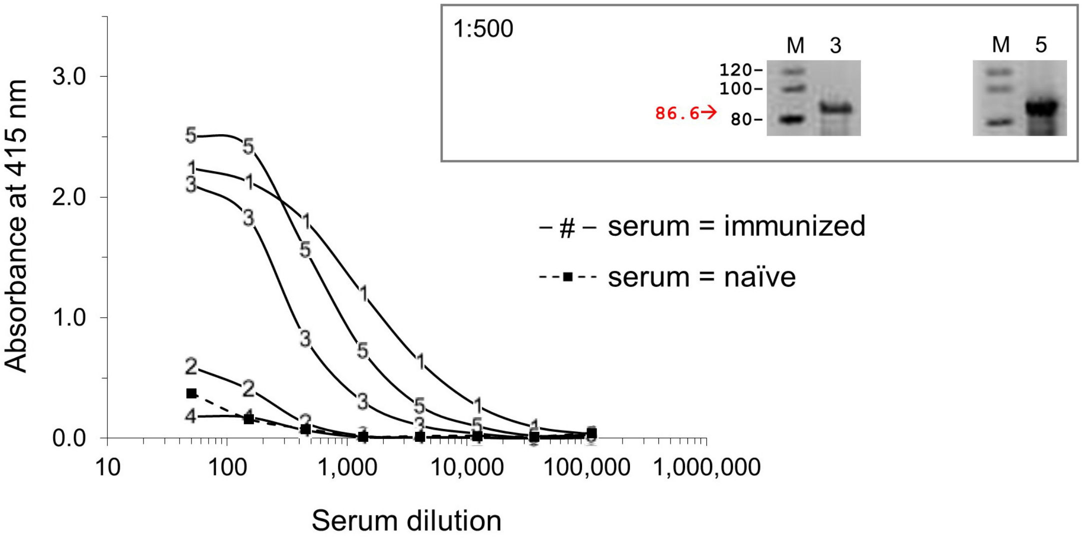

g MPID-030/FTT0919/FupB
3 boosts

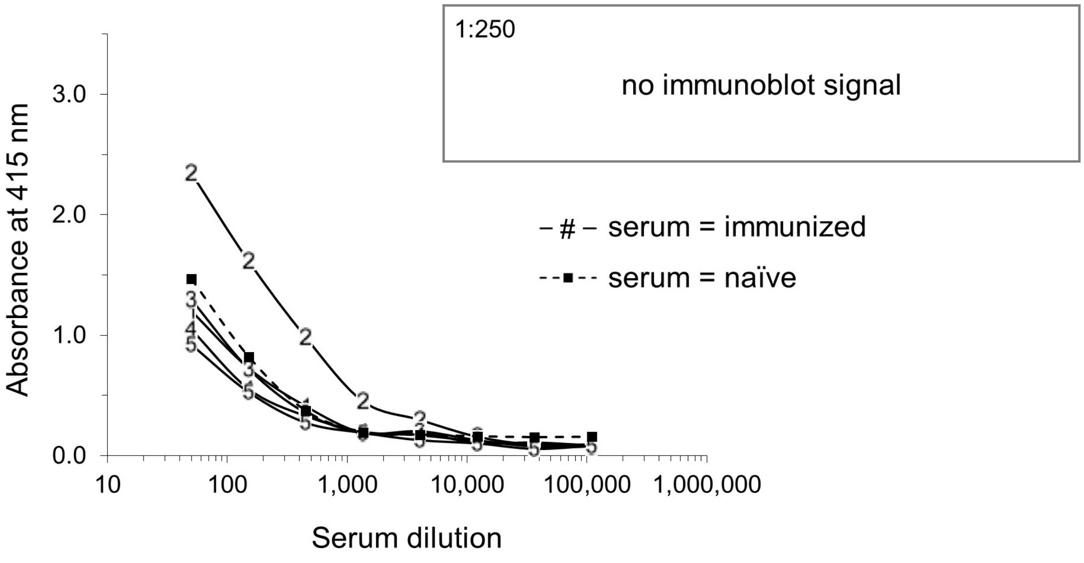

Supplementary Figure 1 (continued)

h MPID-032/FTT0759/hypothetical membrane protein
2 boosts

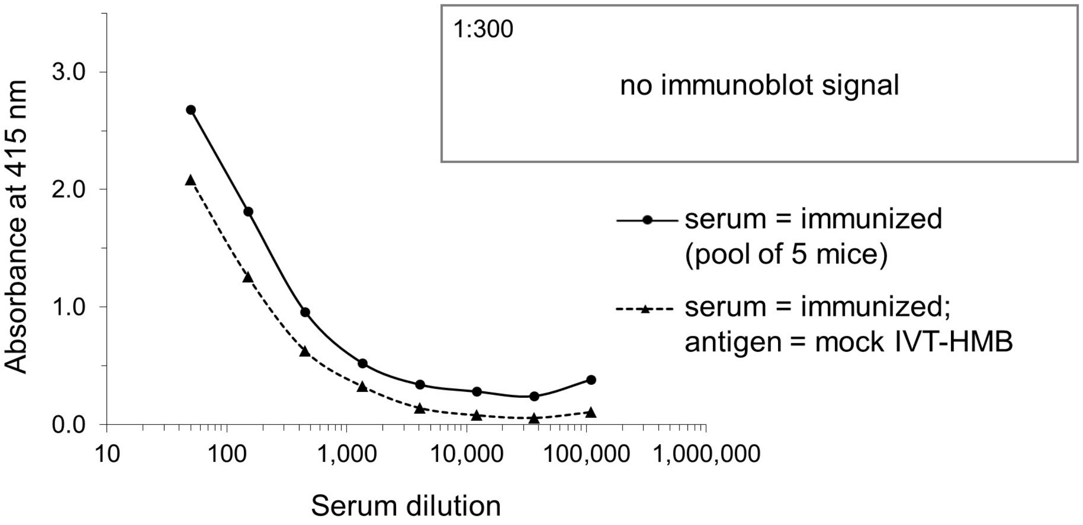

i MPID-033/FTT1416/Flpp3 lipoprotein
2 boosts

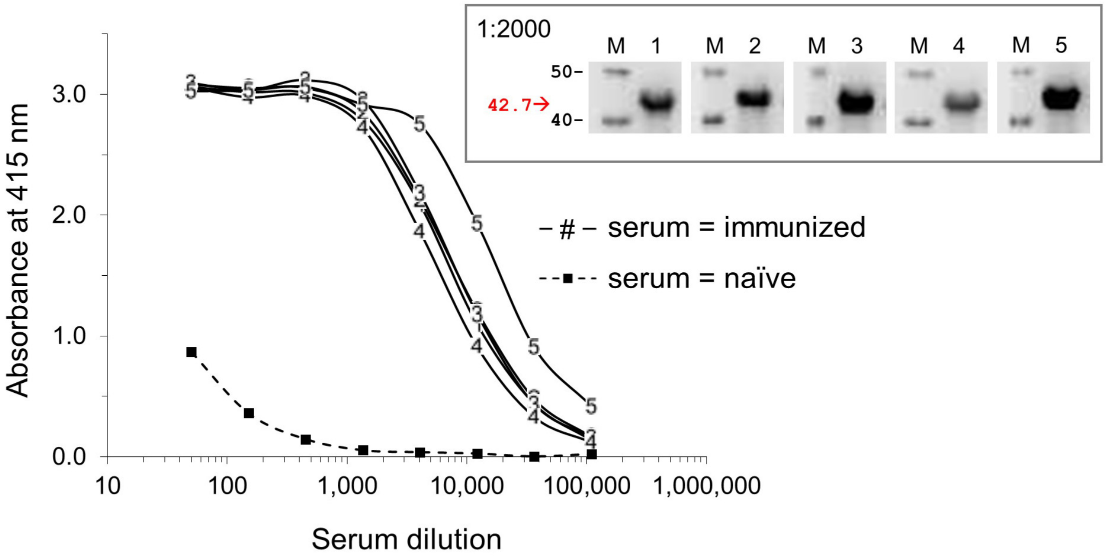

Supplementary Figure 1 (continued)

j MPID-034/FTT1525/hypothetical membrane protein
2 boosts

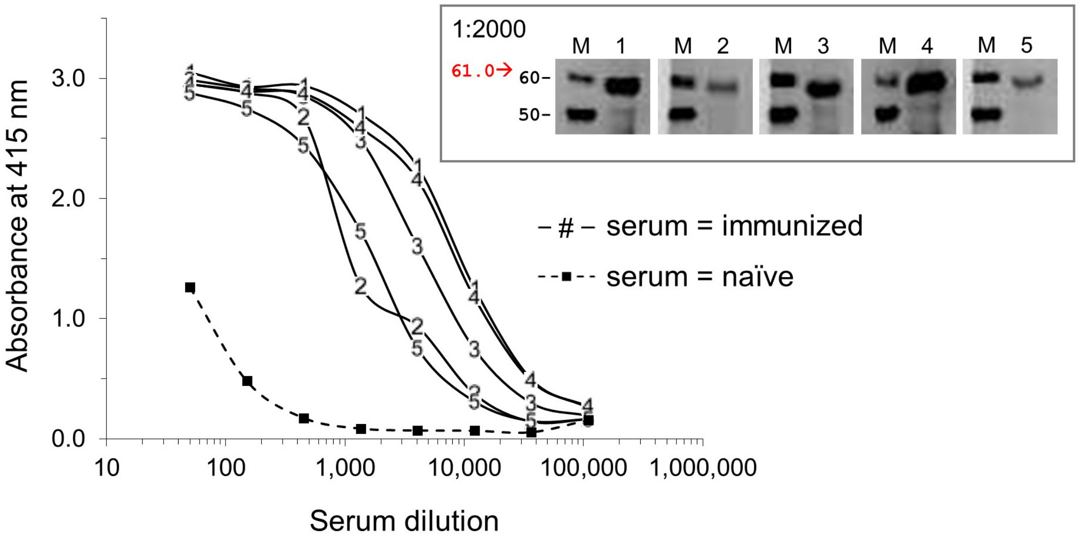

k MPID-035/FTT0807/CapA
4 boosts

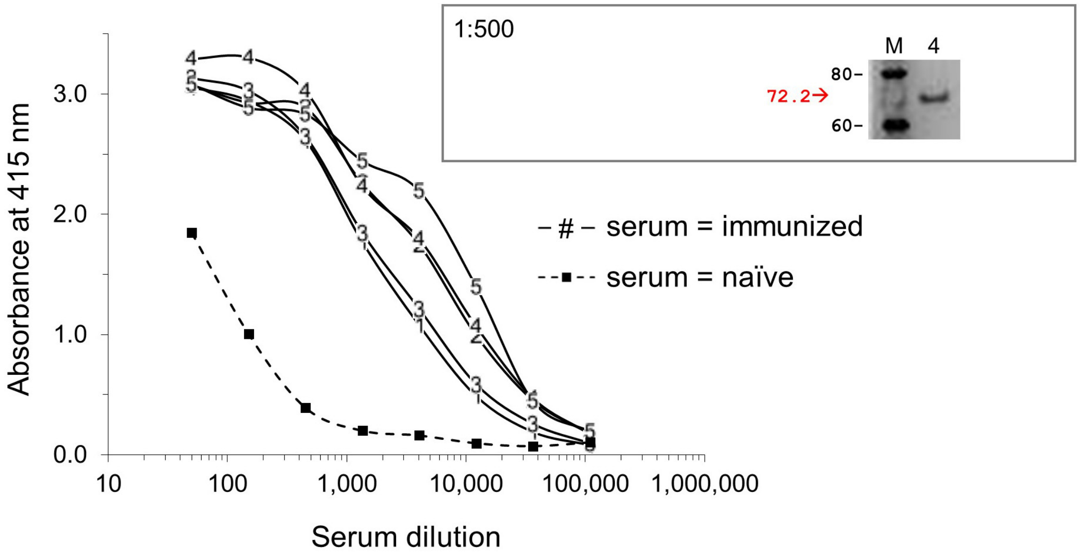

Supplementary Figure 1 (continued)

l MPID-036/FTT0805/CapB
3 boosts

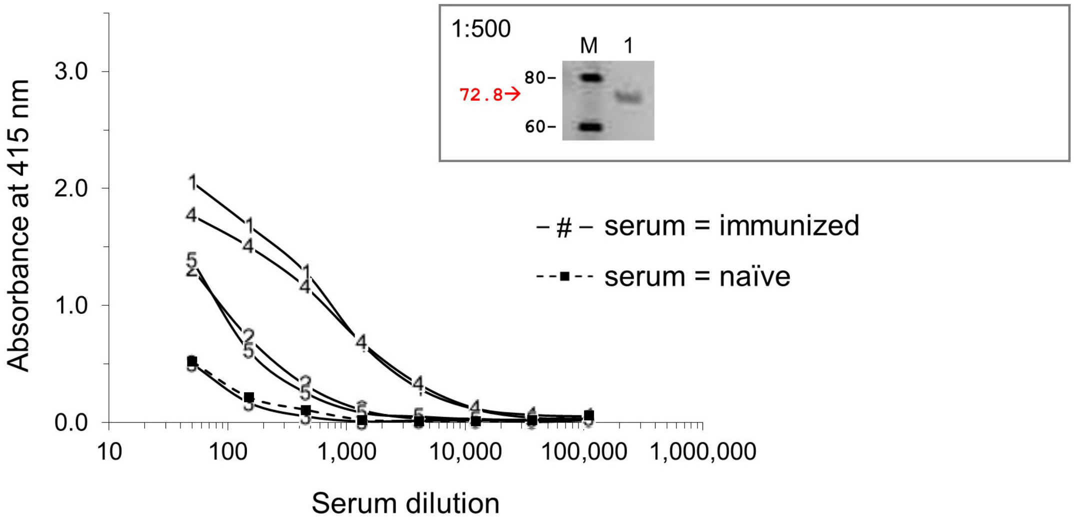

m MPID-037/FTT0806/CapC
2 boosts

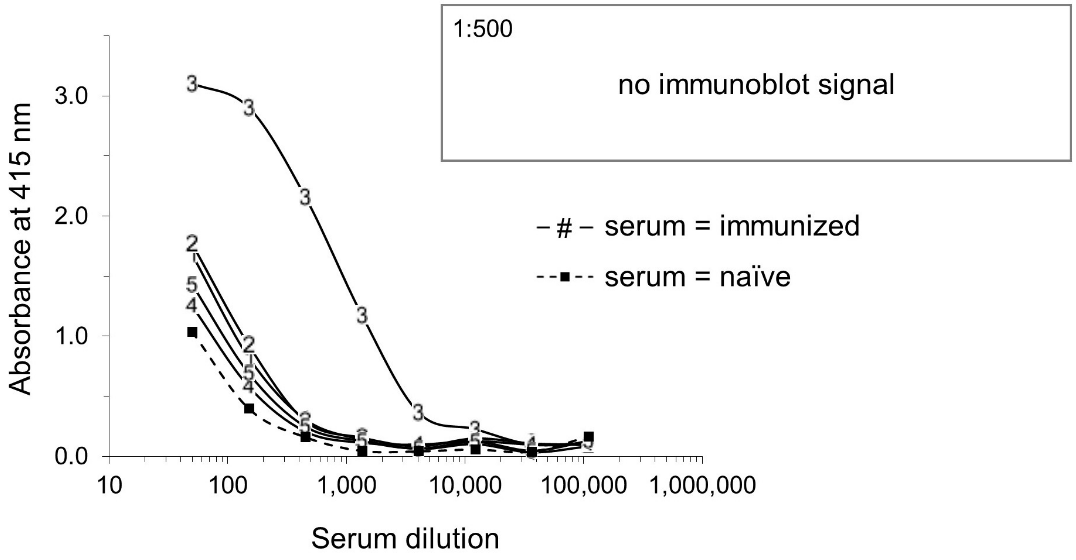

Supplementary Figure 1 (continued)

n MPID-051/FTT1406/hypothetical membrane protein
4 boosts

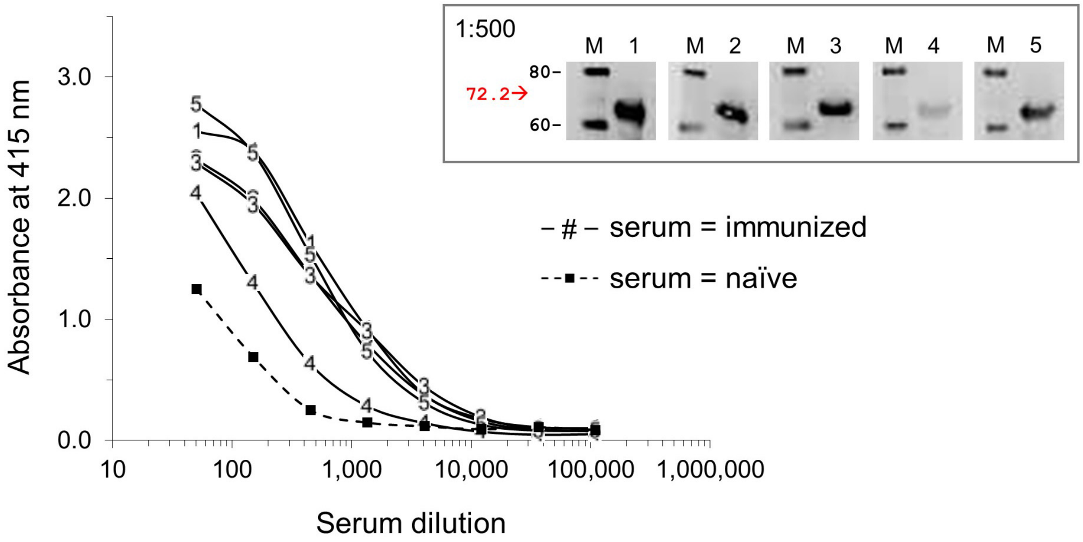

o MPID-021/E183L/p54 envelope protein (Malawi)
4 boosts

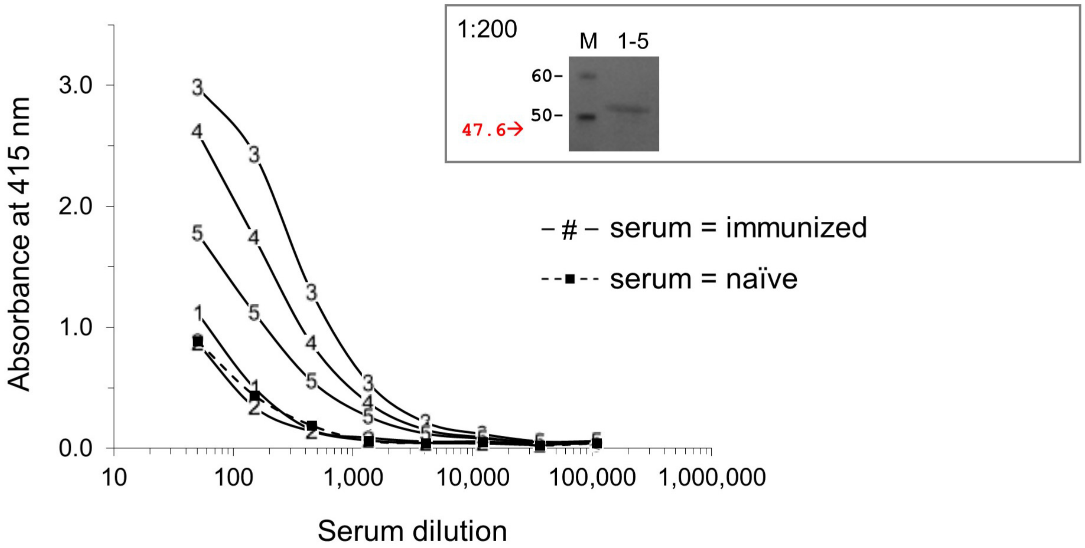

Supplementary Figure 1 (continued)

p MPID-022/EP402R/CD2v
4 boosts

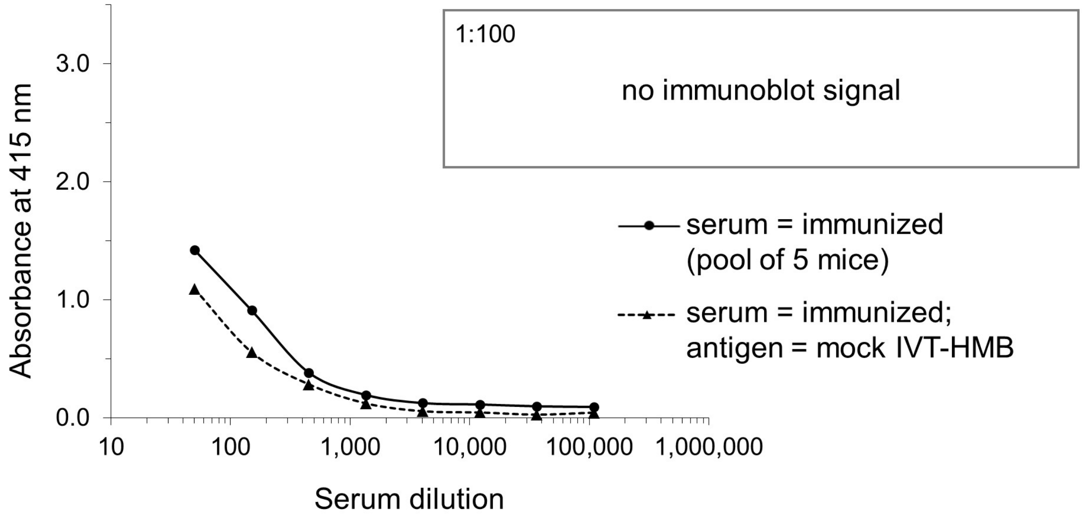

q MPID-023/EP153R/C-type lectin-like protein
4 boosts

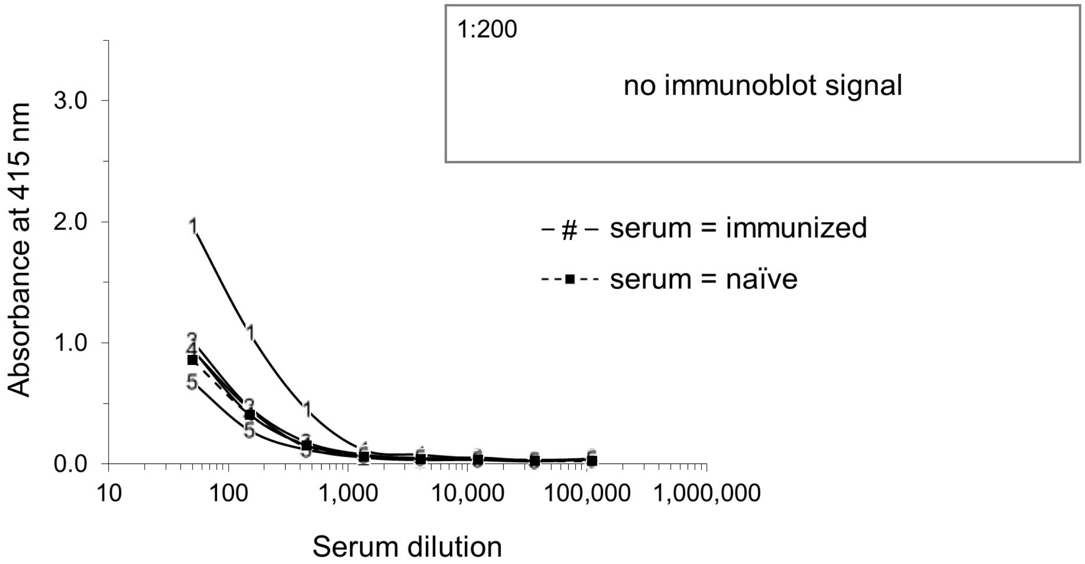

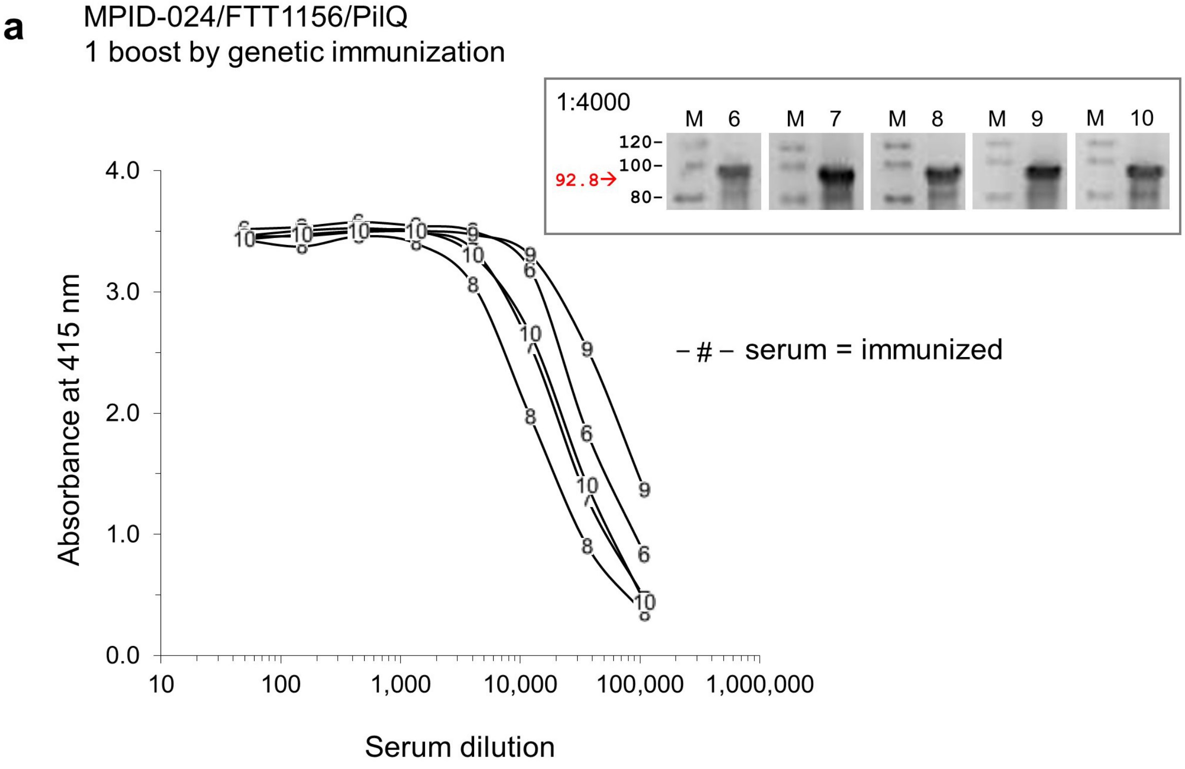

Supplementary Figure 2. Sera reactivity for five targets upon immunization with pCMVi-LSrCOMPTT constructs, plus some IVT-HMB protein boosts. Adjuvants were altered from those in Supplementary Fig. 1 to replace Class C CpG with Class B, and to include pCMVi-LS-LTA-R192G and pCMVi-LTB in all immunization steps. Other details are as described in Supplementary Fig. 1. Numbering of mice is continued from Supplementary Fig. 1. To increase titers after 2 genetic boosts, IVT-HMB protein was injected intraperitoneally as 1 or 2 additional boosts. IVT-HMB protein was derived from pET-32b-TEV constructs (Supplementary Fig. 4), which yields different protein fusion tags from those used in the ELISA and immunoblot analyses. (**a**) PilQ following DNA boost #1. (**b**) OMEP following DNA boost #1, and (**c**) DNA boost #2. (**d**) BamA following DNA boost #1, (**e**) DNA boost #2, and (**f**) IVT-HMB boost #1. (**g**) Flpp3 lipoprotein following DNA boost #1. (**h**) FTT1406 following DNA boost #1, (**i**) DNA boost #2, (**j**) IVT-HMB boost #1, and (**k**) IVT-HMB boost #2. The immunoblot in (**j**) for FTT1406 mouse #10 lacked bands near the expected molecular weight and so was not shown.

Supplementary Figure 2 (continued)

b MPID-025/FTT1258/OMEP
1 boost by genetic immunization

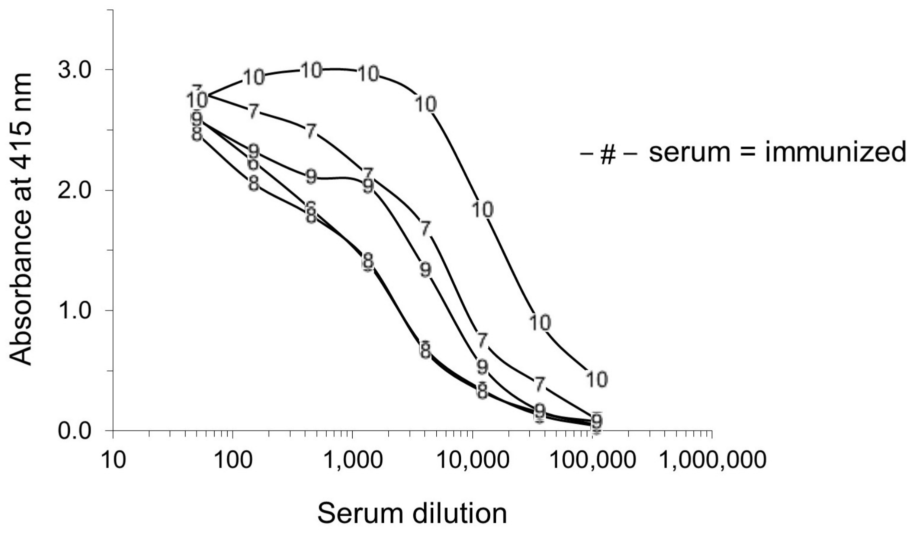

c MPID-025/FTT1258/OMEP
2 boosts by genetic immunization

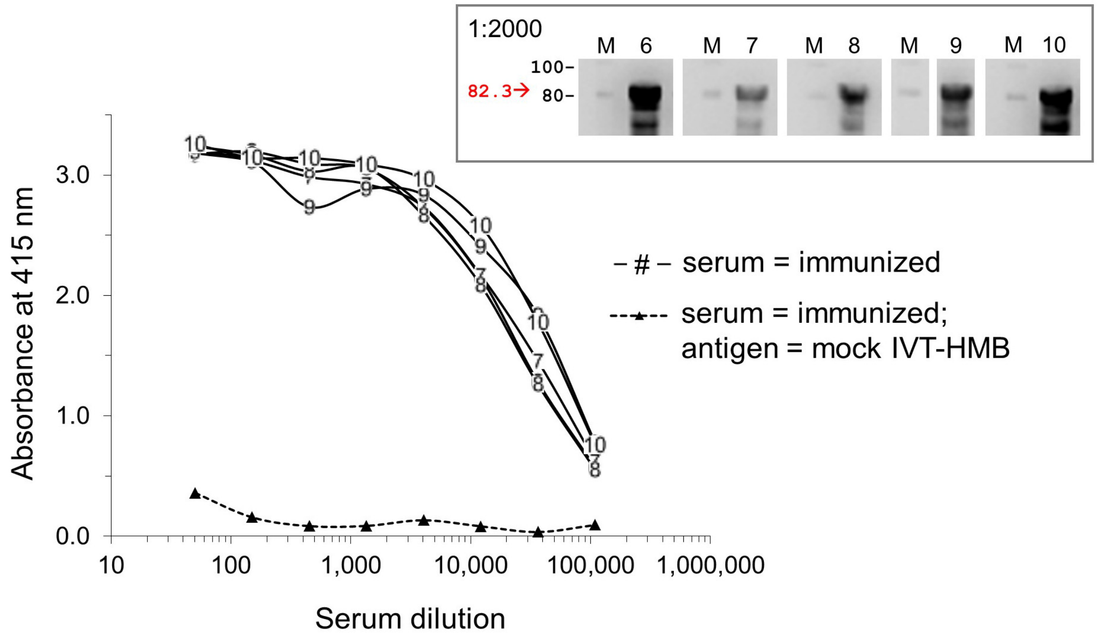

Supplementary Figure 2 (continued)

d MPID-026/FTT1573/BamA
1 boost by genetic immunization

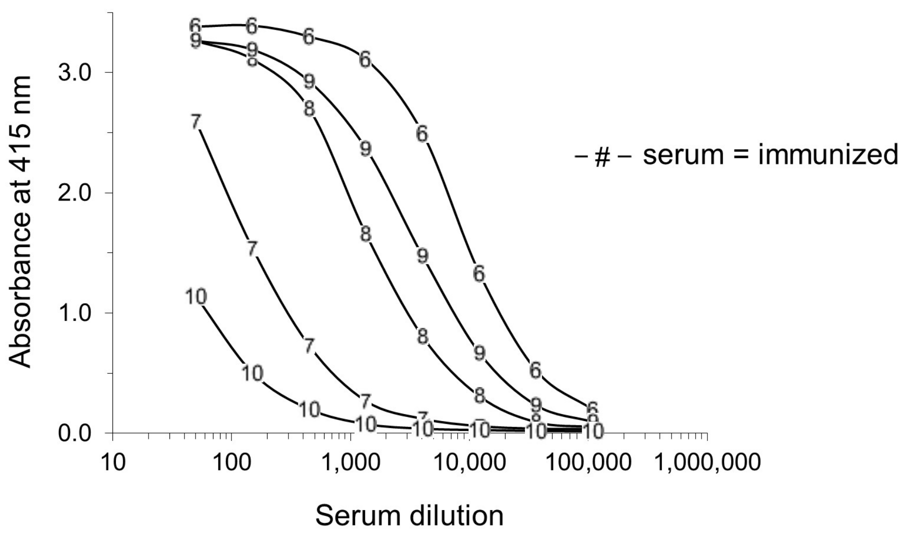

e MPID-026/FTT1573/BamA
2 boosts by genetic immunization

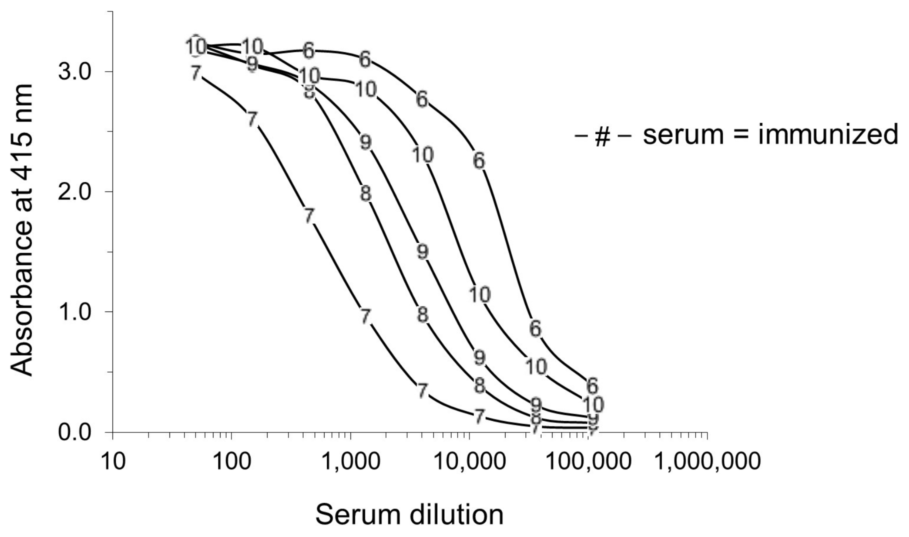

Supplementary Figure 2 (continued)

f MPID-026/FTT1573/BamA
2 boosts by genetic immunization + 1 boost with IVT-HMB

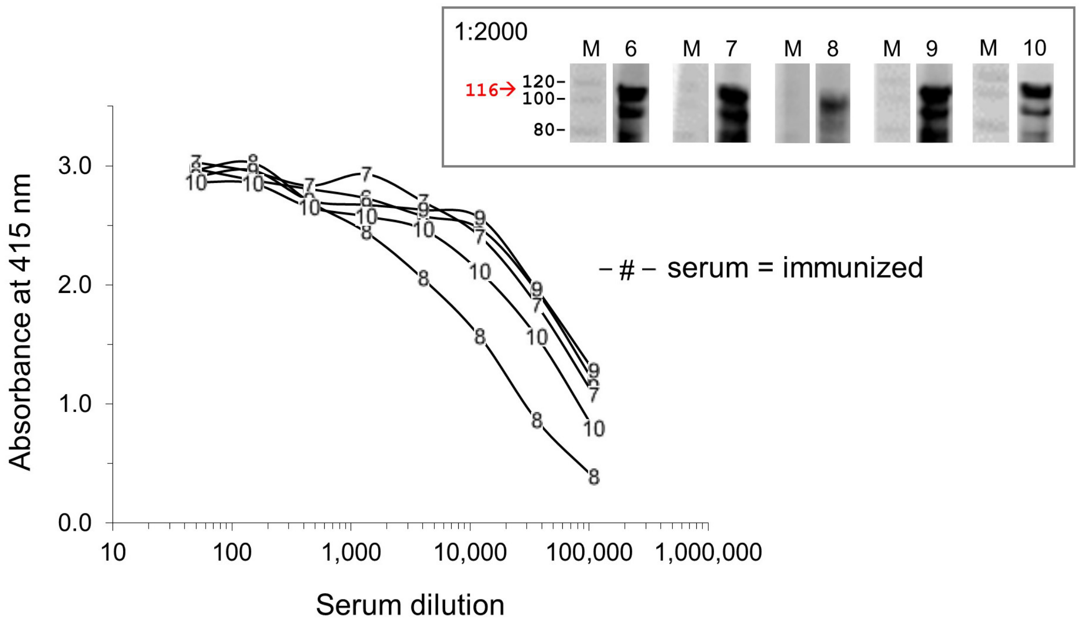

g MPID-033/FTT1416/Flpp3 lipoprotein
1 boost by genetic immunization

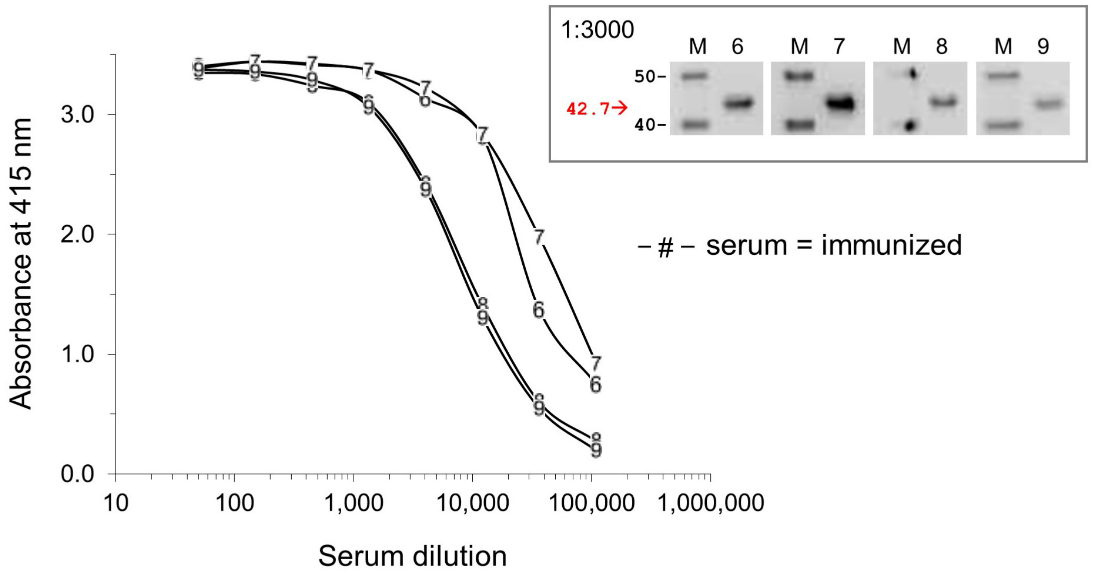

Supplementary Figure 2 (continued)

h MPID-051/FTT1406/hypothetical membrane protein
1 boost by genetic immunization

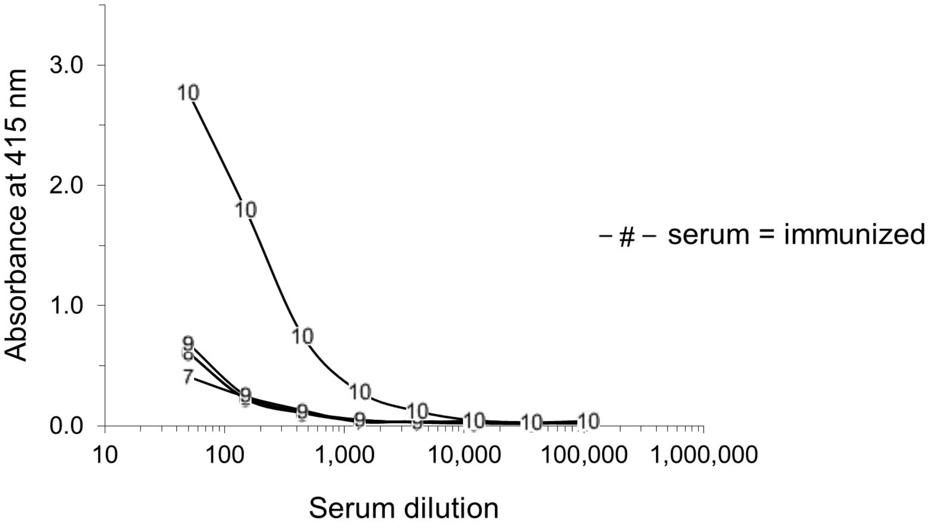

i MPID-051/FTT1406/hypothetical membrane protein
2 boosts by genetic immunization

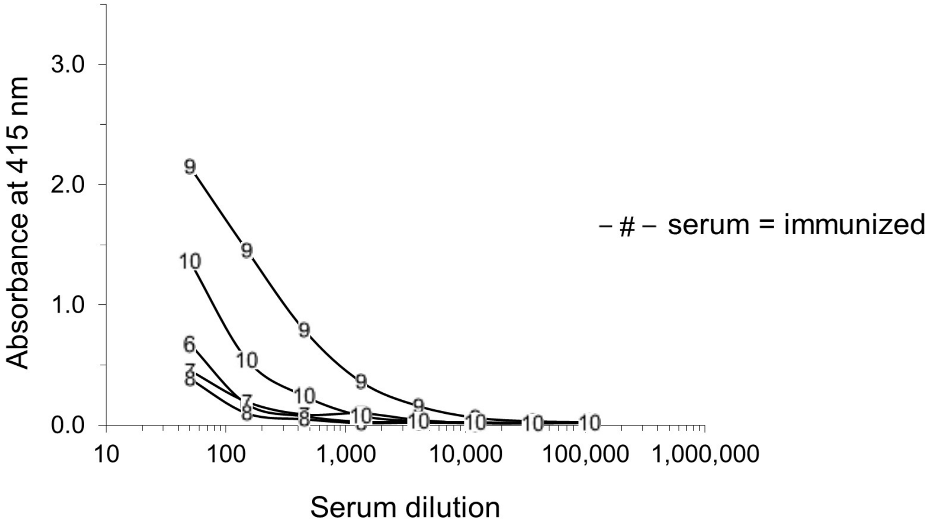

Supplementary Figure 2 (continued)

j MPID-051/FTT1406/hypothetical membrane protein
2 boosts by genetic immunization + 1 boost with IVT-HMB

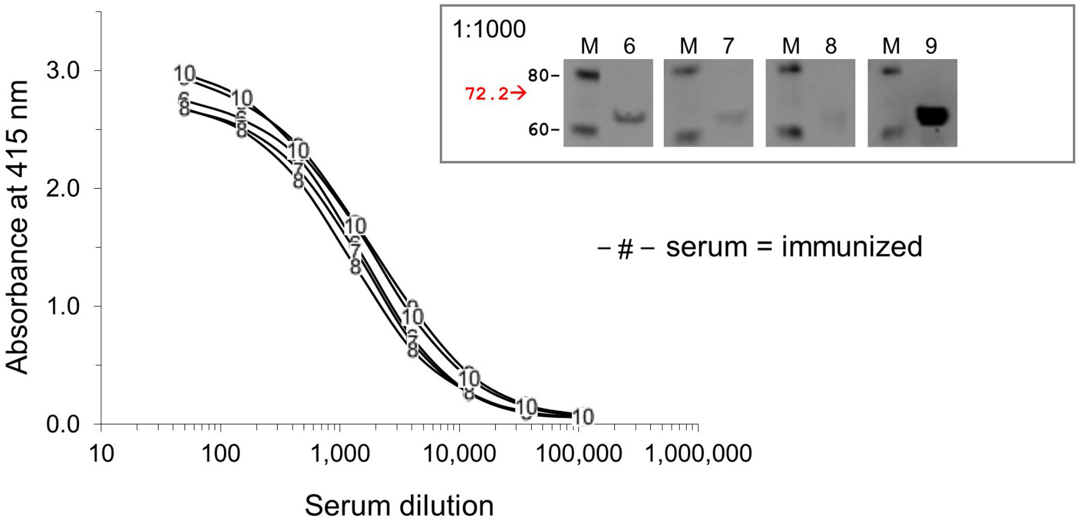

k MPID-051/FTT1406/hypothetical membrane protein
2 boosts by genetic immunization + 2 boosts with IVT-HMB

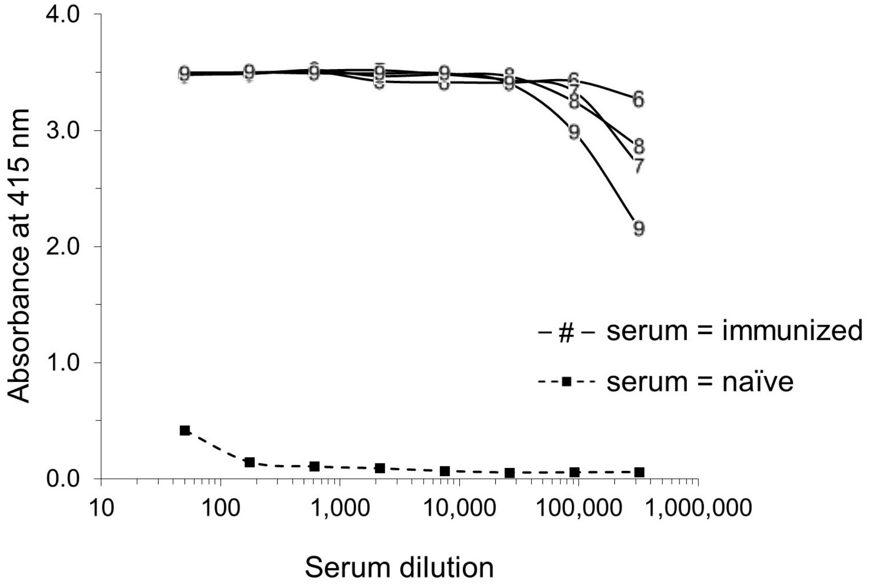

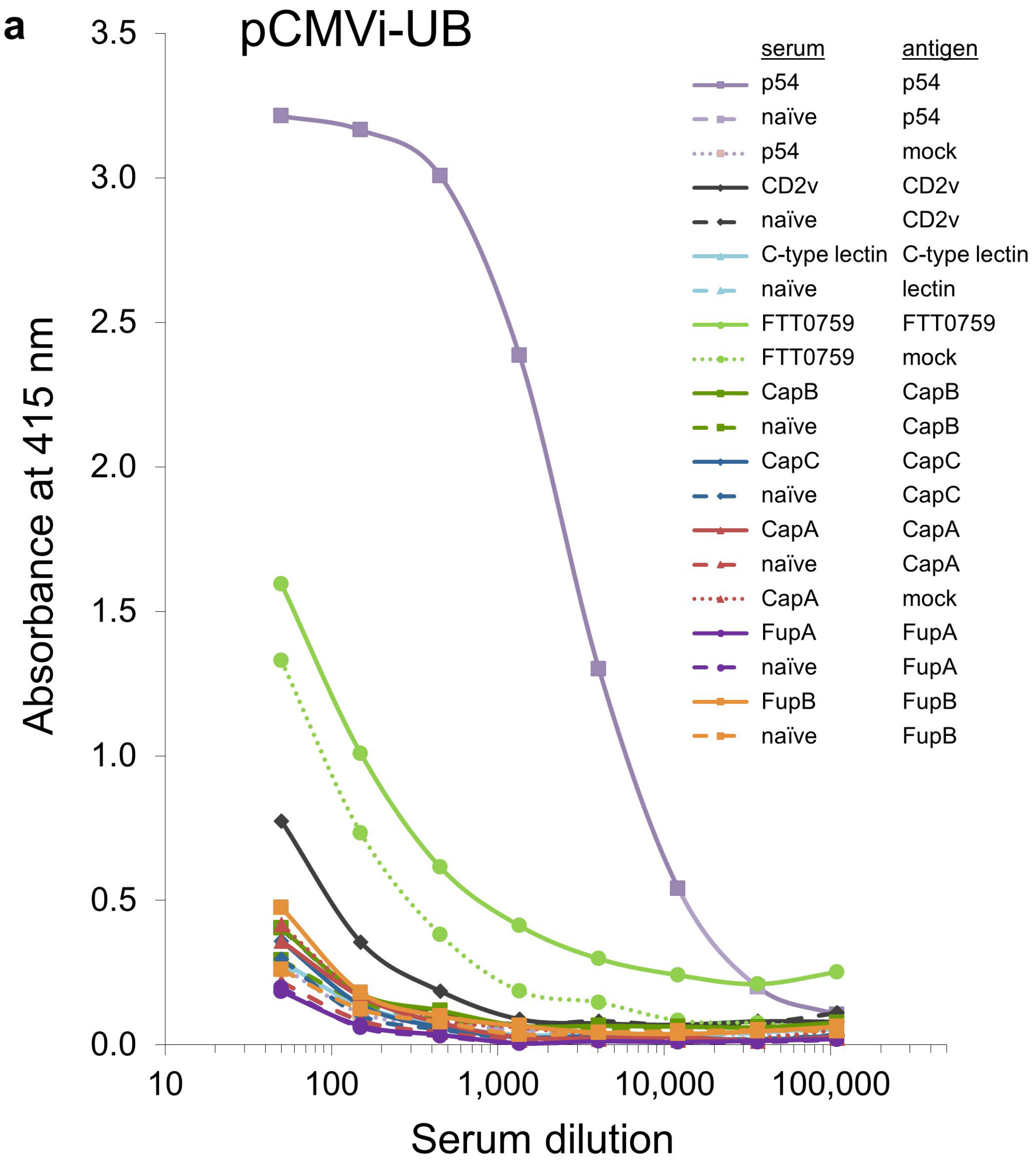

Supplementary Figure 3. Reactivity of sera from mice immunized with pCMVi-UB constructs that encode for nine membrane proteins. Details are as described in Supplementary Fig. 1. Membrane protein targets attempted were only those that yielded little or no sera reactivity upon immunization with pCMVi-LSrCOMPTT constructs (Fig. 2d). (**a**) ELISA results from sera following a single boost by genetic immunization. Sera were analyzed as a pool of equal volumes of serum from the available mice. The legend indicates the corresponding target used to immunize mice ("serum") or added to the ELISA wells in the form of IVT-HMB protein ("antigen"). "Mock" indicates IVT-HMB products from reactions that lacked template DNA. (**b**) Positive immunoblots from five mice immunized with a pCMVi-UB construct containing p54 from the Georgia isolate of ASFV. Sera were obtained following a single boost by genetic immunization.
(**c**) Immunoblot from a pool of sera from these five mice following two additional boosts by intraperitoneal injection of p54 protein obtained from IVT-HMB reactions. Yellow triangles indicate recognition of likely irrelevant proteins. A serum dilution of 1:2000 was used for the immunoblots in (**b**,**c**).

Supplementary Figure 3 (continued)

MPID-021/E183L/p54 envelope protein (Georgia)

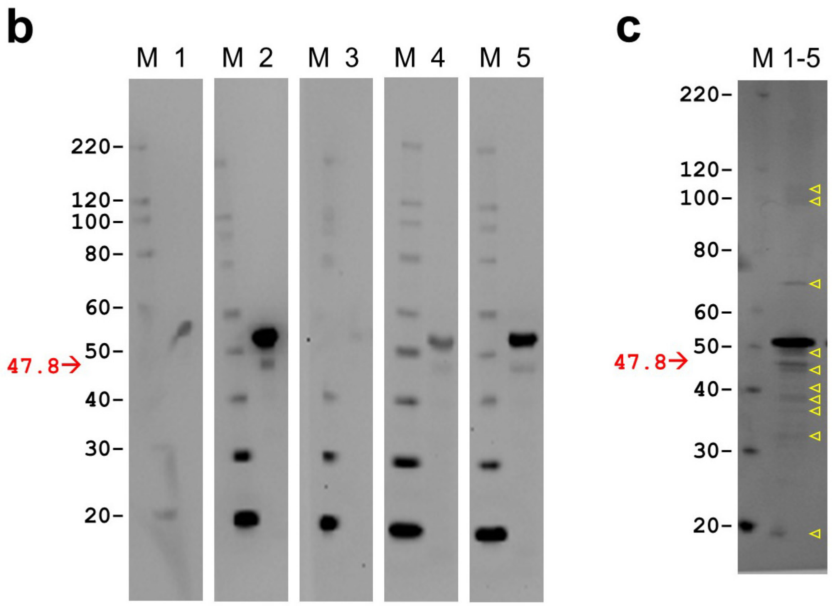

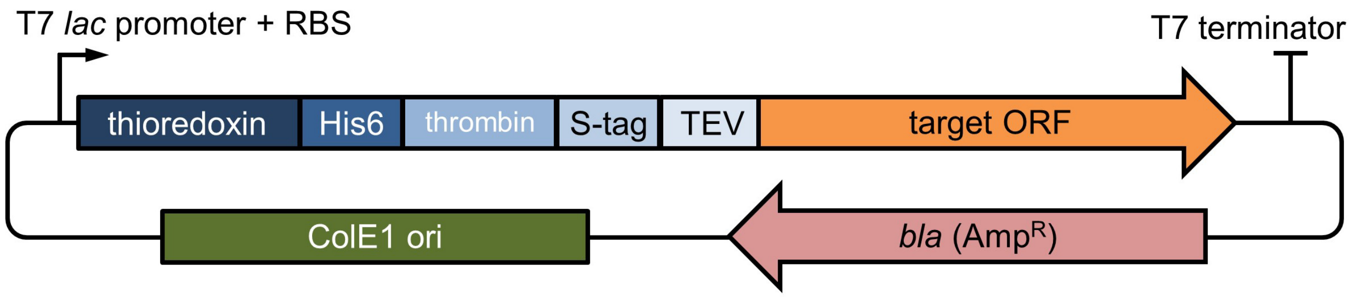

Supplementary Figure 4. Schematic of pET-32b-TEV. This vector was used to express full-length target proteins in IVT-HMB. RBS = ribosome binding site; TEV = Tobacco etch virus protease recognition site.

a

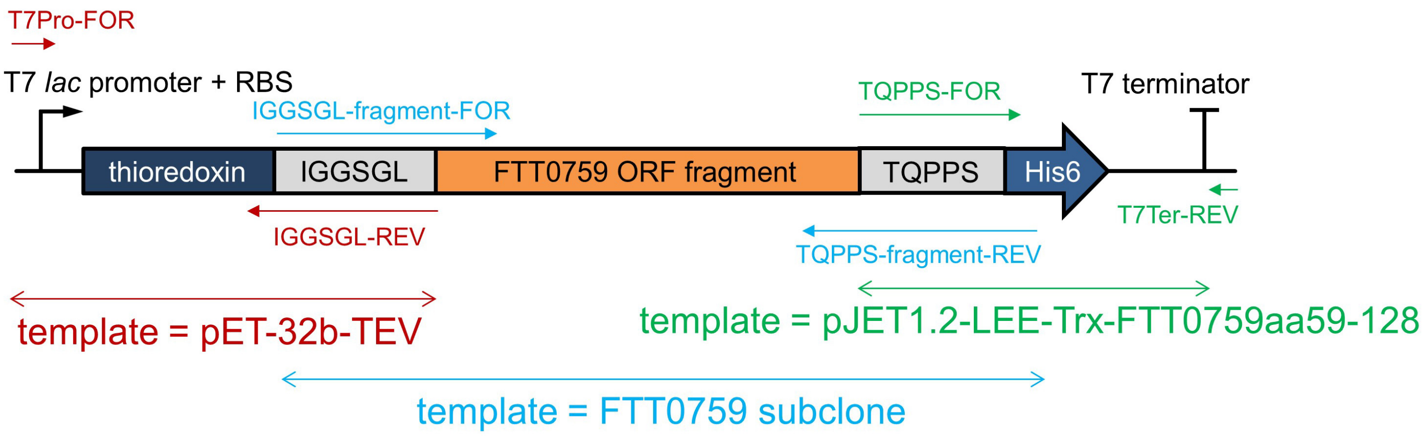

Supplementary Figure 5. Linear expression elements (LEEs) used to express fragments of target proteins. (**a**) General schematic of the LEE[7] DNA constructs for expressing fragments of FTT0759. Colored lines with single and double arrowheads identify the corresponding primers (Supplementary Table 4) and template DNAs (Supplementary Table 2), respectively. RBS = ribosome binding site. Also shown are representative DNA (**b**) and protein (**c**) sequences of the LEE construct for the FTT0759 fragment corresponding to amino acids 59-128. (**d**) General schematic of the LEE DNA constructs for expressing fragments of CD2v. Also shown are representative DNA (**e**) and protein (**f**) sequences of the LEE construct for the CD2v fragment corresponding to amino acids 10-202. Colored DNA sequences in (**b**) and (**e**) correspond to colored protein sequences in (**c**) and (**f**), respectively.

Supplementary Figure 5 (continued)

b

```
LOCUS       LEE\Thio\FTT0759-fragment7-aa59-128         863 bp    DNA     linear       20-FEB-2015
SOURCE
  ORGANISM
FEATURES             Location/Qualifiers
     CDS             96..422
                     /vntifkey="4"
                     /label=thioredoxin
     promoter        8..24
                     /vntifkey="30"
                     /label=T7\promoter
     promoter        27..51
                     /vntifkey="30"
                     /label=lacO
     RBS             79..87
                     /vntifkey="32"
                     /label=RBS
     terminator      792..838
                     /vntifkey="43"
                     /label=T7\terminator
     CDS             441..650
                     /vntifkey="4"
                     /label=FTT0759\fragment\aa\59-128
     mutation        452..452
                     /vntifkey="62"
                     /label=modified\codon\usage,\T\to\C
     mutation        449..449
                     /vntifkey="62"
                     /label=modified\codon\usage,\T\to\C
     CDS             96..683
                     /vntifkey="4"
                     /label=complete\expressed\protein
     misc_feature    666..683
                     /vntifkey="21"
                     /label=His6-tag
BASE COUNT      249 a       190 c       196 g       228 t
ORIGIN
        1 gcgaaattaa tacgactcac tatagggaa ttgtgagcgg ataacaattc ccctctagaa
       61 ataattttgt ttaactttaa gaaggagata tacatatgag cgataaaatt attcacctga
      121 ctgacgacag ttttgacacg gatgtactca aagcggacgg ggcgatcctc gtcgatttct
      181 gggcagagtg gtgcggtccg tgcaaaatga tcgccccgat tctggatgaa atcgctgacg
      241 aatatcaggg caaactgacc gttgcaaaac tgaacatcga tcaaaaccct ggcactgcgc
      301 cgaaatatgg catccgtggt atcccgactc tgctgctgtt caaaaacggt gaagtggcgg
      361 caaccaaagt gggtgcactg tctaaaggtc agttgaaaga gttcctcgac gctaacctgg
      421 ccataggcgg aagcggattg aagacattct tcccacggct taatcttaag caatgggcat
      481 atgtcatcgc tgtaggattt tttggtgtat ttttatacaa tataacgttt ctatgggcgg
      541 aaaaactaat ctcgggtaat attgttgcta ttatctatgc ttttactcct tgcttaataa
      601 caattttatc tagctatatt tttaatctca aagtaaatca acaagctaaa acccaacctc
      661 cctcccacca tcatcatcat cattaataaa agggcgaatt ccagcacact ggcggccgtt
      721 actagtggat ccggctgcta acaaagcccg aaaggaagct gagttggctg ctgccaccgc
      781 tgagcaataa ctagcataac cccttggggc ctctaaacgg gtcttgaggg gttttttgct
      841 gaaaggagga actatatccg gat
//
```

c

```
LOCUS       Translation\of\LEE\Thio\FTT0759-fragment7-aa59-128\         196 aa                          20-FEB-2015
DEFINITION  Translation of a fragment of LEE Thio FTT0759-fragment7-aa59-128.
KEYWORDS    TRANSLATED.
SOURCE
  ORGANISM
FEATURES             Location/Qualifiers
     Region          1..109
                     /vntifkey="1000"
                     /label=thioredoxin
     Region          116..185
                     /vntifkey="1000"
                     /label=FTT0759-fragment7-aa59-128
     Site            191..196
                     /vntifkey="264"
                     /label=His6-tag
ORIGIN
        1 msdkiihltd dsfdtdvlka dgailvdfwa ewcgpckmia pildeiadey qgkltvakln
       61 idqnpgtapk ygirgiptll lfkngevaat kvgalskgql kefldanlai ggsglktffp
      121 rlnlkqwayv iavgffgvfl ynitflwaek lisgnivaii yaftpcliti lssyifnlkv
      181 nqqaktqpps hhhhhh
```

Supplementary Figure 5 (continued)

d

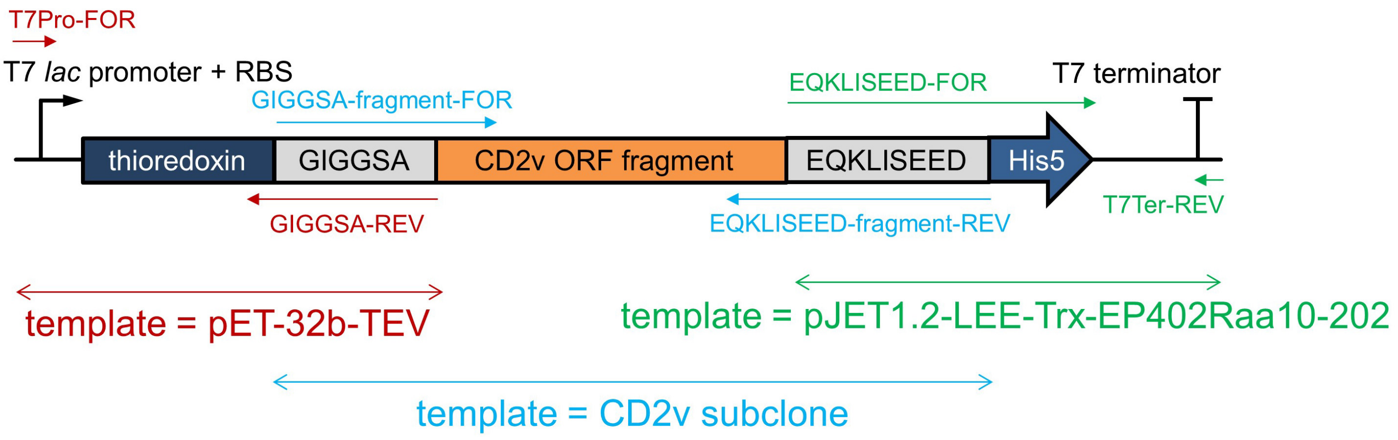

Supplementary Figure 5 (continued)

e

```
LOCUS       LEE EP402R_MPID-022_CD2v_aa10-202_Thio        1241 bp    DNA     linear        20-FEB-2015
SOURCE
  ORGANISM
FEATURES             Location/Qualifiers
     promoter        8..24
                     /vntifkey="30"
                     /label=T7\promoter
     CDS             96..422
                     /vntifkey="4"
                     /label=thioredoxin
     misc_feature    1047..1061
                     /vntifkey="21"
                     /label=His5-tag
     RBS             79..87
                     /vntifkey="32"
                     /label=RBS
     CDS             441..1019
                     /vntifkey="4"
                     /label=CD2v\fragment\aa\10-202
     terminator      1170..1216
                     /vntifkey="43"
                     /label=T7\terminator
     promoter        27..51
                     /vntifkey="30"
                     /label=lacO
     CDS             96..1061
                     /vntifkey="4"
                     /label=complete\expressed\protein
BASE COUNT      434 a       213 c       227 g       367 t
ORIGIN
        1 gcgaaattaa tacgactcac tatagggaa ttgtgagcgg ataacaattc ccctctagaa
       61 ataattttgt ttaactttaa gaaggagata tacatatgag cgataaaatt attcacctga
      121 ctgacgacag ttttgacacg gatgtactca aagcggacgg ggcgatcctc gtcgatttct
      181 gggcagagtg gtgcggtccg tgcaaaatga tcgccccgat tctggatgaa atcgctgacg
      241 aatatcaggg caaactgacc gttgcaaaac tgaacatcga tcaaaaccct ggcactgcgc
      301 cgaaatatgg catccgtggt atcccgactc tgctgctgtt caaaaacggt gaagtggcgg
      361 caaccaaagt gggtgcactg tctaaaggtc agttgaaaga gttcctcgac gctaacctgg
      421 ccggtatagg cggaagcgcc tctaacatag ttttaagtat tgattattgg gttagtttta
      481 ataaaacaat aattttagat agtaatatta ctaatgataa taatgatata aatggagtat
      541 catggaattt ttttaataat tcttttaata cactagctac atgtggaaaa gcaggtaact
      601 tttgtgaatg ttctaattat agtacatcaa tatataatat aacaaataat tgtagcttaa
      661 ctatttttcc tcataatgat gtatttgata caacatatca agtagtatgg aatcaaataa
      721 ttaattatac aataaaatta ttaacacctg ctactccccc aaatatcaca tataattgta
      781 ctaatttttt aataacatgt aaaaaaaata atggaacaaa cactaatata tatttaaata
      841 taaatgatac ttttgttaaa tatactaatg aaagtatact tgaatataac tggaataata
      901 gtaacattaa caattttaca gctacatgta taattaataa tacaattagt acatctaatg
      961 aaacaacact tataaattgt acttatttaa cattgtcatc taactatttt tatacttttg
     1021 aacagaaact gattagcgaa gaagaccatc atcatcatca ttaataaaag ggcgaattcc
     1081 agcacactgg cggccgttac tagtggatcc ggctgctaac aaagcccgaa aggaagctga
     1141 gttggctgct gccaccgctg agcaataact agcataaccc cttggggcct ctaaacgggt
     1201 cttgaggggt ttttgctga aaggaggaac tatatccgga t
//
```

f

```
LOCUS       Translation\of\ LEE EP402R_MPID-022_CD2v_aa10-202_Thio        322 aa                      20-FEB-2015
DEFINITION  Translation of a fragment of LEE EP402R_MPID-022_CD2v_aa10-202_Thio.
KEYWORDS    TRANSLATED.
SOURCE
  ORGANISM
FEATURES             Location/Qualifiers
     Region          1..109
                     /vntifkey="1000"
                     /label=thioredoxin
     Region          116..308
                     /vntifkey="1000"
                     /label= CD2v\fragment\aa\10-202
     Site            318..322
                     /vntifkey="264"
                     /label=His5-tag
ORIGIN
        1 msdkiihltd dsfdtdvlka dgailvdfwa ewcgpckmia pildeiadey qgkltvakln
       61 idqnpgtapk ygirgiptll lfkngevaat kvgalskgql kefldanlag iggsasnivl
      121 sidywvsfnk tiildsnitn dnndingvsw nffnnsfntl atcgkagnfc ecsnystsiy
      181 nitnncslti fphndvfdtt yqvvwnqiin ytiklltpat ppnitynctn flitckkng
      241 tntniylnin dtfvkytnes ileynwnnsn innftatcii nntistsnet tlinctyltl
      301 ssnyfytfeq kliseedhhh hh
//
```

SUPPLEMENTARY TABLES

Supplementary Table 1. Target membrane proteins in this study: immunogenicity, physical characteristics, and role in pathogenesis.

Source	MPID no. [a]	Locus [b]	Accession no.	Protein name [b]	Polyclonal antibody by genetic immunization [e]	Reported immunogenicity	Molecular weight (kDa)	β-barrel protein (β) or no. of α-helical transmembrane domains (α) [r]	Hydrophobicity [s]	Putative membrane localization [t]	Reported role in pathogenesis
Francisella tularensis SCHU S4	024	FTT1156	YP_170123	PilQ	+	+[f,g]	65	β	36%	OM	
	025	FTT1258	YP_170216	OMEP, outer membrane efflux protein	+	+[f]	55	β	37%	OM	
	026	FTT1573	YP_170495	BamA/YaeT	+	+[f]	88	β	35%	OM	
	027	FTT1724	YP_170626	TolC	+	+[f,h]	57	β	36%	OM	+[v]
	028	FTT0583	YP_169607	FopA	+	+[f,g,h,i,j,k,l]	42	β	34%	OM	+[w,x,y,z]
	029	FTT0918	YP_169915	FupA [c]	±	+[f,i,m,n]	59	β	35%	OM lipo [u]	+[w,aa]
	030	FTT0919	YP_169916	FupB [c]	-	+[f]	53	β	38%	OM	
	032	FTT0759	YP_169769	hypothetical	-		34	9-10α	54%	IM	+[w]
	033	FTT1416	YP_170359	Flpp3 lipoprotein [c]	+	+[h]	15	0-1α	31%	OM lipo	+[w]
	034	FTT1525	YP_170455	Hypothetical	+		33	2α	38%	IM	+[w]
	035	FTT0807	YP_169814	CapA [c]	±		44	2α	36%	IM	+[w,bb]
	036	FTT0805	YP_169812	CapB	±		45	1α	38%	IM	+[w,cc]
	037	FTT0806	YP_169813	CapC	-		17	4-5α	53%	IM	+[w,bb]
	051	FTT1406	YP_170350	hypothetical	+	+[h]	44	2-3α	45%	IM	
African swine fever virus	021	E183L	Q89784	p54 envelope protein (Malawi) [d]	±	+[o]	19	1α	33%	IE	+[dd]
	021	E183L	CAQ53724	p54 envelope protein (Georgia) [d]	+	+[o]	19	1α	33%	IE	+[dd]
	022	EP402R	CBW46724	CD2v (Georgia) [d]	-	+[p]	41	3-4α	31%	OE	+[ee]
	023	EP153R	CBW46723	C-type lectin-like (Georgia) [d]	-	±[q]	18	1α	30%	ER	+[ff]

Supplementary Table 1 (continued)

[a] MPID number designation is according to the Protein Structure Initiative[10].

[b] FTT numbers and protein names are from[11].

[c] FupA, FupB protein designations are from[12], Flpp3 is from[13], and CapA is from[14].

[d] Malawi is the ASFV Malawi Lil 20/1 isolate[15], and Georgia is the ASFV Georgia 2007/1 isolate[16]. p54 (Malawi) and p54 (Georgia) have 87% identity.

[e] This work. Polyclonal antibodies were produced by genetic immunization and were scored as follows: +, positive immunoblot obtained for 5 out of 5 mice at ≥1:500 dilution; ±, positive immunoblot obtained for less than 5 out of 5 mice or at ≤1:500 dilution; -, no reactivity by immunoblot.

[f] Polyclonal antibodies were generated in rats injected with purified denatured recombinant protein[17].

[g] The target protein was identified by mass spectrometry of a membrane-enriched protein fraction from the *F. tularensis* live vaccine strain (LVS) run on 2D-PAGE, upon probing with sera from mice immunized with *F. tularensis* LVS[18].

[h] Proteome microarray signal suggested the presence of target-specific antibody in sera of mice immunized with killed *F. tularensis* LVS[19].

[i] Protein was identified in immunoblots of 2D-PAGE containing total protein from *F. tularensis* strain FSC033, using sera of mice immunized with *F. tularensis* LVS and subsequently challenged with *F. tularensis* FSC033[20].

[j] Protein was identified by mass spectrometry of a membrane-enriched protein fraction from *F. tularensis* LVS run on 2D-PAGE, upon probing with sera from patients diagnosed with tularemia[21].

[k] Two-dimensional gel electrophoresis, immunoblotting, and mass spectrometry analyses revealed an antibody response to this protein in mice experimentally infected with *F. tularensis* LVS, and in human patients diagnosed with tularemia, but not in control sera from mice or humans[22].

[l] Monoclonal antibodies against FopA were isolated following sublethal infection with *F. tularensis* LVS plus boosting with sonicated bacteria[23].

[m] Protein was identified in immunoblots using sera from a lab assistant who was accidently infected with *F. tularensis* SCHU S4[24].

[n] Polyclonal antibodies were raised upon immunization of guinea pigs with a recombinantly-expressed, GST-tagged fragment (amino acids 25-176) of FupA[12].

[o] Western analyses with sera from convalescent pigs indicate that p54 is one of the most highly antigenic proteins in the ASFV proteome[25,26].

[p] Evidence for immunogenicity of the post-translationally modified (glycosylated) CD2v is a ~75 kDa band that was detected in immunoblots of protein from baculovirus-infected Sf cells expressing CD2v from ASFV isolate $E75CV_1$ using pig sera following infection with ASFV strain 1207VR11[27]. A similarly-sized band was also detected in protein from extracellular particles of ASFV strain BA71V that were isolated from an infected Vero cell line, using pig sera following immunizations with CD2v-expressing baculovirus-infected Sf cells[27]. Host glycosylation of CD2v effectively doubles this protein's apparent molecular weight[28].

[q] Rabbit serum with low reactivity (1:100 serum dilution) was reported following peptide immunization[29].

[r] Shown are the number of α-helical transmembrane domains as predicted using the programs TopPred[30], TMHMM[31], SOSUI[32], and TMpred[33].

[s] Percentage total by weight of amino acid residues A, F, I, L, V, and W.

[t] Putative localization to the outer membrane (OM) or inner membrane (IM) is based on protein designations[11], predicted transmembrane domains (this work), and/or physical determinations[17,34]. Localization of lipoproteins (lipo) to the inner or outer membrane is predicted by the second amino acid

following the lipidation site, as has been characterized in *E. coli*[35]. p54 localizes to the inner envelope (IE) of ASFV[36]; CD2v to the outer envelope (OE) of ASFV[37]; and the C-type lectin to the endoplasmic reticulum (ER) of an infected cell line[38].

[u] Lipoprotein designation was predicted by [17,39,40].

[v] Deletion of *tolC* in *F. tularensis* LVS caused significant attenuation of virulence in a mouse model[41] and led to increased secretion of proinflammatory cytokines. TolC delays activation of the intrinsic apoptotic pathway during infection of primary macrophages and during organ colonization in a mouse model[42].

[w] The target protein was indicated as a virulence determinant in *F. tularensis* LVS by signature-tagged mutagenesis and subsequent infection of a mouse model[43].

[x] Passive transfer of anti-FopA antibodies allowed 40% survival of mice infected with *F. tularensis* LVS[23].

[y] FopA-specific antibodies protected against lethal intradermal and intranasal challenges with *F. tularensis* LVS but not *F. tularensis* SCHU S4[44].

[z] Msutagenesis of FopA in *Francisella novicida* strain U112[45] and in *F. tularensis* strain SCHU S4[46] led to attenuated growth in macrophages.

[aa] Mutation of *fupA* attenuated mouse infection by *F. tularensis* SCHU S4[12]. FupA is also required for high-affinity ferrous iron uptake[12].

[bb] In a whole-genome screen of *F. novicida* strain U112, mutation of *capA* or *capC* led to loss of colonization of the spleen in an inhalation mouse model[47].

[cc] Deletion of *capB* significantly attenuated infection in either *F. tularensis* SCHU S4[48] or *F. tularensis* LVS[49].

[dd] p54 mediates specific binding to macrophages[50], recruitment of the endoplasmic reticulum membrane[36,51], and binding to dynein, the light chain of the microtubule motor protein[52,53].

[ee] CD2v has roles in hemadsorption[54,55] and immunosuppression[56].

[ff] The C-type lectin is implicated in anti-apoptosis[57], hemadsorption[29], and interaction with the host MHC I[38].

Supplementary Table 2. Plasmid vectors used in this study.

Plasmid name	DNASU Plasmid ID[58]	Usage	Reference
Empty parent vectors			
pCMVi-LSrCOMPTT	EvNO00629005	Genetic immunization	This study
pCMVi-UB	EvNO00629006	Genetic immunization	59
pCMVi-LS	EvNO00631283	Genetic immunization	59
pRSET-natGFP	EvNO00623703	GFP-tagged expression	14
pRSET-natGFPHis	EvNO00623704	GFP-His-tagged expression	14
pET-32b-TEV	EvNO00629003	Thioredoxin-tagged expression	This study
pCDF-BAD	EvNO00631285	Arabinose-inducible expression in *E. coli*	This study
Accessory vectors for genetic immunization			
pCMVi-LS-LTA-R192G	EcCD00697199	DNA adjuvant for genetic immunization	60
pCMVi-LS-LTB	EcCD00697200	DNA adjuvant for genetic immunization	60
Subclones			
FTT0583 in pCMVi-LSrCOMPTT	FtCD00665310	Genetic immunization	This study
FTT0759 in pCMVi-LSrCOMPTT	FtCD00665313	Genetic immunization	This study
FTT0805 in pCMVi-LSrCOMPTT	FtCD00665317	Genetic immunization	This study
FTT0806 in pCMVi-LSrCOMPTT	FtCD00665321	Genetic immunization	This study
FTT0807 in pCMVi-LSrCOMPTT	FtCD00665324	Genetic immunization	This study
FTT0918 in pCMVi-LSrCOMPTT	FtCD00696809	Genetic immunization	This study
FTT0919 in pCMVi-LSrCOMPTT	FtCD00665293	Genetic immunization	This study
FTT1156 in pCMVi-LSrCOMPTT	FtCD00696813	Genetic immunization	This study
FTT1258 in pCMVi-LSrCOMPTT	FtCD00665297	Genetic immunization	This study
FTT1406 in pCMVi-LSrCOMPTT	FtCD00696704	Genetic immunization	This study
FTT1416 in pCMVi-LSrCOMPTT	FtCD00665299	Genetic immunization	This study
FTT1525 in pCMVi-LSrCOMPTT	FtCD00665303	Genetic immunization	This study
FTT1573 in pCMVi-LSrCOMPTT	FtCD00696814	Genetic immunization	This study
FTT1724 in pCMVi-LSrCOMPTT	FtCD00697212	Genetic immunization	This study
E183L in pCMVi-LSrCOMPTT	AsCD00665311	Genetic immunization	This study
EP153R in pCMVi-LSrCOMPTT	AsCD00665318	Genetic immunization	This study
EP402R in pCMVi-LSrCOMPTT	AsCD00665314	Genetic immunization	This study
EP402R in pCMVi-LS	AsCD00665308	Genetic immunization	This study
FTT0759 in pCMVi-UB	FtCD00670281	Genetic immunization	This study
FTT0805 in pCMVi-UB	FtCD00670287	Genetic immunization	This study
FTT0806 in pCMVi-UB	FtCD00670290	Genetic immunization	This study
FTT0807 in pCMVi-UB	FtCD00670293	Genetic immunization	This study
FTT0918 in pCMVi-UB	FtCD00696802	Genetic immunization	This study
FTT0919 in pCMVi-UB	FtCD00696804	Genetic immunization	This study
E183L in pCMVi-UB	AsCD00670301	Genetic immunization	This study
EP153R in pCMVi-UB	AsCD00670311	Genetic immunization	This study
EP402R in pCMVi-UB	AsCD00670306	Genetic immunization	This study
FTT0583 in pET-32b-TEV	FtCD00696807	Thioredoxin-tagged expression	This study
FTT0759 in pET-32b-TEV	FtCD00697211	Thioredoxin-tagged expression	This study

FTT0805 in pET-32b-TEV	FtCD00696798	Thioredoxin-tagged expression	This study
FTT0806 in pET-32b-TEV	FtCD00670280	Thioredoxin-tagged expression	This study
FTT0807 in pET-32b-TEV	FtCD00670285	Thioredoxin-tagged expression	This study
FTT0918 in pET-32b-TEV	FtCD00696799	Thioredoxin-tagged expression	This study
FTT0919 in pET-32b-TEV	FtCD00696800	Thioredoxin-tagged expression	This study
FTT1156 in pET-32b-TEV	FtCD00696801	Thioredoxin-tagged expression	This study
FTT1258 in pET-32b-TEV	FtCD00696803	Thioredoxin-tagged expression	This study
FTT1406 in pET-32b-TEV	FtCD00670299	Thioredoxin-tagged expression	This study
FTT1416 in pET-32b-TEV	FtCD00670304	Thioredoxin-tagged expression	This study
FTT1525 in pET-32b-TEV	FtCD00696806	Thioredoxin-tagged expression	This study
FTT1573 in pET-32b-TEV	FtCD00696808	Thioredoxin-tagged expression	This study
FTT1724 in pET-32b-TEV	FtCD00670317	Thioredoxin-tagged expression	This study
E183L in pET-32b-TEV	AsCD00696703	Thioredoxin-tagged expression	This study
EP153R in pET-32b-TEV	AsCD00670286	Thioredoxin-tagged expression	This study
EP402R in pET-32b-TEV	AsCD00697195	Thioredoxin-tagged expression	This study
FTT0583 in pRSET-natGFP	FtCD00697197	GFP-tagged expression	This study
FTT0759 in pRSET-natGFP	FtCD00670296	GFP-tagged expression	This study
FTT0805 in pRSET-natGFP	FtCD00696717	GFP-tagged expression	This study
FTT0806 in pRSET-natGFP	FtCD00670303	GFP-tagged expression	This study
FTT0807 in pRSET-natGFP	FtCD00670309	GFP-tagged expression	This study
FTT0918 in pRSET-natGFP	FtCD00696720	GFP-tagged expression	This study
FTT0919 in pRSET-natGFP	FtCD00670316	GFP-tagged expression	This study
FTT1156 in pRSET-natGFP	FtCD00696702	GFP-tagged expression	This study
FTT1258 in pRSET-natGFP	FtCD00670279	GFP-tagged expression	This study
FTT1406 in pRSET-natGFP	FtCD00670284	GFP-tagged expression	This study
FTT1416 in pRSET-natGFP	FtCD00670288	GFP-tagged expression	This study
FTT1525 in pRSET-natGFP	FtCD00670292	GFP-tagged expression	This study
FTT1573 in pRSET-natGFP	FtCD00696713	GFP-tagged expression	This study
FTT1724 in pRSET-natGFP	FtCD00670297	GFP-tagged expression	This study
E183L in pRSET-natGFPHis	AsCD00670283	GFP-His-tagged expression	This study
E183L in pRSET-natGFP	AsCD00670298	GFP-tagged expression	This study
EP153R in pRSET-natGFP	AsCD00670310	GFP-tagged expression	This study
EP402R in pRSET-natGFP	AsCD00697198	GFP-tagged expression	This study
pJET-LEE-Thio-FTT0759aa59-128	FtCD00697194	PCR template for generation of LEE DNA	This study
pJET-LEE-Thio-EP402Raa10-202	AsCD00697924	PCR template for generation of LEE DNA	This study
pRSET-FTT0583	FtCD00665298	Expression of the tag-free target in *E. coli*	This study
pRSET-FTT1156	FtCD00665309	Expression of the tag-free target in *E. coli*	This study
pRSET-FTT1258	FtCD00665312	Expression of the tag-free target in *E. coli*	This study
pRSET-FTT1525	FtCD00665315	Expression of the tag-free target in *E. coli*	This study
pCDF-BAD-EcBamA-FTT1573	FtCD00696810	Expression of the tag-free target in *E. coli*	This study

Supplementary Table 3. Representative oligonucleotide primers used to generate subclones.

Subclone name	Forward and reverse primer sequences (5' to 3') [a]
pCDF-BAD-EcBamA-FTT1573	AAGGAGATATACAT*ATGGCGATGAAAAAGTTGCTCATAG*<br>GCGCCCTCGAGCATA***CTA****TTAGAAATTTTGTCCTAATGAGAATTG*
pRSET-FTT1156 (tagless)	GAAGGAGATATACAT*ATGTTTTTTGCAAAAAAGAAGTTATTAGTTGTTC*<br>CGGAACCTGCTGATCC***TCA****TTTTTGGACTCTTGACTCAATGATTTTTG*
FTT1156 in pCMVi-LSrCOMPTT	GGTAGCGGAGATCTG*ATGTTTTTTGCAAAAAAGAAGTTATTAGTTGTTC*<br>GCCATGCAAGCTTAT***TCA****TTTTTGGACTCTTGACTCAATGATTTTTG*
E183L in pCMVi-UB	CGCCTGAGAGGTGCA*ATGGATTCTGAATTTTTTCAACCGGTTTATC*<br>ATGCCACCCGGGATC**CTA***CAAGGAGTTTTCTAGGTCTTTATGC*
FTT1156 in pET-32b-TEV	GCGATATCGGATCCG*ATGTTTTTTGCAAAAAAGAAGTTATTAGTTGTTC*<br>CGGCCGCAAGCTT***TCA****TTTTTGGACTCTTGACTCAATGATTTTTG*
FTT1156 in pRSET-natGFP	GAAGGAGATATACAT*ATGTTTTTTGCAAAAAAGAAGTTATTAGTTGTTC*<br>CGGAACCTGCTGATCC*TTTTTGGACTCTTGACTCAATGATTTTTG*

[a] Primers were designed for ligation-independent cloning as described in the Methods. Underlined sequence corresponds to the parent vector, italicized sequence corresponds to the membrane protein, and bold sequence indicates a stop codon.

Supplementary Table 4. Oligonucleotide primers used for construction of LEEs.

Primer name [a]	Sequence (5' to 3') [b]
Primers for LEE fragments flanking the ORF	
T7Pro-FOR	GCGAAATTAATACGACTCACTATAGG
GIGGSA-REV	GGCGCTTCCGCCTATACCGGCCAGGTTAGCGTCGAGG
IGGSGL-REV	CAATCCGCTTCCGCCTAT
T7Ter-REV	ATCCGGATATAGTTCCTCCTTTCAG
EQKLISEED-FOR	GAACAGAAACTGATTAGCGAAGAAGACCATCATCATCATCATTAATAAAAGGGCG
TQPPS-FOR	ACCCAACCTCCCTCCCAC
IGGSGL-fragment-FOR primers	
FTT0759aa1F	ATAGGCGGAAGCGGATTG*ATGAACTCTTCATTAACAAAAGGCTACG*
FTT0759aa149F	ATAGGCGGAAGCGGATTG*GCGGAATGCTTAAATGGTGTTCAG*
FTT0759aa59F	ATAGGCGGAAGCGGATTG_AAGACATTC*TTC*CCACGGCTTAATC_ [c]
FTT0759aa179F	ATAGGCGGAAGCGGATTG*AAGTGTTGTGTTAGAGAAGGTGTAC*
FTT0759aa120F	ATAGGCGGAAGCGGATTG*AATCTCAAAGTAAATCAACAAGCTAAAATTGG*
TQPPS-fragment-REV primers	
FTT0759aa158R	GGAGGGAGGTTGGGT*GTTAATCTGAACACCATTTAAGCATTCC*
FTT0759aa305R	GGAGGGAGGTTGGGT*ATTGCCAGACTTACGATTAACTGCAG*
FTT0759aa250R	GGAGGGAGGTTGGGT*ACCTATTTCTTCAAGAGCTCCTAAATAC*
FTT0759aa220R	GGAGGGAGGTTGGGT*ATCGGTATATGCTAACTGAGAAAAATCAG*
FTT0759aa128R	GGAGGGAGGTTGGGT*TTTAGCTTGTTGATTTACTTTGAGATTAAAAATATAG*
FTT0759aa192R	GGAGGGAGGTTGGGT*ATTTATTGTGATCATGTGTACACCTTCTC*
GIGGSA-fragment-FOR primers	
CD2vaa10F	GGTATAGGCGGAAGCGCC*TCTAACATAGTTTTAAGTATTGATTATTGGGTTAG*
CD2vaa170F	GGTATAGGCGGAAGCGCC*GCTACATGTATAATTAATAATACAATTAGTACATC*
EQKLISEED-fragment-REV primers	
CD2vaa202R	GTCTTCTTCGCTAATCAGTTTCTGTTC*AAAAGTATAAAAATAGTTAGATGACAATGTTAAATAAG*
CD2vaa355R	GTCTTCTTCGCTAATCAGTTTCTGTTC*GTGAATAAGCGAAATATTTTGGGTAGATAATG*

[a] Coloring corresponds to that used in Supplementary Fig. 5a,d. The underlined protein sequences in the left column of the table correspond to the underlined primer sequences in the right column of the table.

[b] Italicized sequences correspond to FTT0759 or CD2v.

[c] "C*" indicates modification from a T nucleotide in order to adjust the T_m of the primer. The protein sequence was not affected.

SUPPLEMENTARY REFERENCES

1. Pawlita, M., Mushinski, E., Feldmann, R. J. & Potter, M. A monoclonal antibody that defines an idiotope with two subsites in galactan-binding myeloma proteins. *J. Exp. Med.* **154**, 1946-1956 (1981).
2. Kawashima, I. *et al.* Mouse monoclonal antibodies detecting disialogangliosides on mouse and human T lymphomas. *Int. J. Cancer* **41**, 267-274 (1988).
3. Vieira, J. G., Federico, P., Matsueda, G. & Neer, R. M. Monoclonal antibodies to bovine parathyroid hormone: production and characterization. *Braz. J. Med. Biol. Res.* **21**, 1005-1011 (1988).
4. Bennick, A., Grøn, B. & Brosstad, F. Hybridomas can successfully be prepared from frozen/thawn spleen cells. *Hybridoma* **10**, 761-765 (1991).
5. Marusich, M. F. Efficient hybridoma production using previously frozen splenocytes. *J. Immunol. Methods* **114**, 155-159 (1988).
6. Poonam, M. & Butchaiah, G. Production of hybridoma cell lines secreting antibodies to bovine herpesvirus-I using previously frozen splenocytes. *J. Appl. Anim. Res.* **14**, 65-73 (1998).
7. Sykes, K. F. & Johnston, S. A. Linear expression elements: a rapid, *in vivo*, method to screen for gene functions. *Nat. Biotechnol.* **17**, 355-359 (1999).
8. Rath, A., Glibowicka, M., Nadeau, V. G., Chen, G. & Deber, C. M. Detergent binding explains anomalous SDS-PAGE migration of membrane proteins. *Proc. Natl. Acad. Sci. U.S.A.* **106**, 1760-1765 (2009).
9. Leo, J. C., Oberhettinger, P. & Linke, D. Assessing the outer membrane insertion and folding of multimeric transmembrane β-barrel proteins. *Methods Mol. Biol.* **1329**, 157-167 (2015).
10. Gifford, L. K., Carter, L. G., Gabanyi, M. J., Berman, H. M. & Adams, P. D. The Protein Structure Initiative Structural Biology Knowledgebase Technology Portal: a structural biology web resource. *J. Struct. Funct. Genomics* **13**, 57-62 (2012).
11. Larsson, P. *et al.* The complete genome sequence of *Francisella tularensis*, the causative agent of tularemia. *Nat. Genet.* **37**, 153-159 (2005).
12. Ramakrishnan, G., Sen, B. & Johnson, R. Paralogous outer membrane proteins mediate uptake of different forms of iron and synergistically govern virulence in *Francisella tularensis tularensis*. *J. Biol. Chem.* **287**, 25191-25202 (2012).
13. Parra, M. C. *et al.* Identification, cloning, expression, and purification of *Francisella* lpp3: an immunogenic lipoprotein. *Microbiol. Res.* **165**, 531-545 (2010).
14. Martin-Garcia, J. M. *et al.* Purification and biophysical characterization of the CapA membrane protein FTT0807 from *Francisella tularensis*. *Biochemistry* **53**, 1958-1970 (2014).
15. Dixon, L. K. Molecular cloning and restriction enzyme mapping of an African swine fever virus isolate from Malawi. *J. Gen. Virol.* **69**, 1683-1694 (1988).
16. Rowlands, R. J. *et al.* African swine fever virus isolate, Georgia, 2007. *Emerg. Infect. Dis.* **14**, 1870-1874 (2008).
17. Huntley, J. F., Conley, P. G., Hagman, K. E. & Norgard, M. V. Characterization of *Francisella tularensis* outer membrane proteins. *J. Bacteriol.* **189**, 561-574 (2007).
18. Twine, S. M., Petit, M. D., Fulton, K. M., House, R. V. & Conlan, J. W. Immunoproteomics analysis of the murine antibody response to vaccination with an improved *Francisella tularensis* live vaccine strain (LVS). *PLoS One* **5**, e10000 (2010).
19. Eyles, J. E. *et al.* Immunodominant *Francisella tularensis* antigens identified using proteome microarray. *Proteomics* **7**, 2172-2183 (2007).
20. Twine, S. M. *et al.* Immunoproteomic analysis of the murine antibody response to successful and failed immunization with live anti-*Francisella* vaccines. *Biochem. Biophys. Res. Commun.* **346**, 999-1008 (2006).
21. Janovská, S. *et al.* Identification of immunoreactive antigens in membrane proteins enriched fraction from *Francisella tularensis* LVS. *Immunol. Lett.* **108**, 151-159 (2007).
22. Havlasová, J. *et al.* Proteomic analysis of anti-*Francisella tularensis* LVS antibody response in murine model of tularemia. *Proteomics* **5**, 2090-2103 (2005).

23. Savitt, A. G., Mena-Taboada, P., Monsalve, G. & Benach, J. L. *Francisella tularensis* infection-derived monoclonal antibodies provide detection, protection, and therapy. *Clin. Vaccine Immunol.* **16**, 414-422 (2009).
24. Janovská, S. *et al.* Proteomic analysis of antibody response in a case of laboratory-acquired infection with *Francisella tularensis* subsp. *tularensis*. *Folia Microbiol. (Praha)* **52**, 194-198 (2007).
25. Alcaraz, C. *et al.* Highly specific confirmatory Western blot test for African swine fever virus antibody detection using the recombinant virus protein p54. *J. Virol. Methods* **52**, 111-119 (1995).
26. Gómez-Puertas, P. *et al.* Neutralizing antibodies to different proteins of African swine fever virus inhibit both virus attachment and internalization. *J. Virol.* **70**, 5689-5694 (1996).
27. Ruiz-Gonzalvo, F., Rodríguez, F. & Escribano, J. M. Functional and immunological properties of the baculovirus-expressed hemagglutinin of African swine fever virus. *Virology* **218**, 285-289 (1996).
28. Goatley, L. C. & Dixon, L. K. Processing and localization of the African swine fever virus CD2v transmembrane protein. *J. Virol.* **85**, 3294-3305 (2011).
29. Galindo, I., Almazán, F., Bustos, M. J., Viñuela, E. & Carrascosa, A. L. African swine fever virus EP153R open reading frame encodes a glycoprotein involved in the hemadsorption of infected cells. *Virology* **266**, 340-351 (2000).
30. Claros, M. G. & von Heijne, G. TopPred II: an improved software for membrane protein structure predictions. *Comput. Appl. Biosci.* **10**, 685-686 (1994).
31. Sonnhammer, E. L., von Heijne, G. & Krogh, A. A hidden Markov model for predicting transmembrane helices in protein sequences. *Proc. Int. Conf. Intell. Syst. Mol. Biol.* **6**, 175-182 (1998).
32. Hirokawa, T., Boon-Chieng, S. & Mitaku, S. SOSUI: classification and secondary structure prediction system for membrane proteins. *Bioinformatics* **14**, 378-379 (1998).
33. Hofmann, K. & Stoffel, W. TMbase - a database of membrane spanning proteins segments. *Biol. Chem. Hoppe Seyler* **347**, 166 (1993).
34. Dresler, J., Klimentova, J. & Stulik, J. *Francisella tularensis* membrane complexome by blue native/SDS-PAGE. *J. Proteomics* **75**, 257-269 (2011).
35. Masuda, K., Matsuyama, S. & Tokuda, H. Elucidation of the function of lipoprotein-sorting signals that determine membrane localization. *Proc. Natl. Acad. Sci. U.S.A.* **99**, 7390-7395 (2002).
36. Rodríguez, J. M., García-Escudero, R., Salas, M. L. & Andrés, G. African swine fever virus structural protein p54 is essential for the recruitment of envelope precursors to assembly sites. *J. Virol.* **78**, 4299-1313 (2004).
37. Dixon, L. K. *et al.* in Virus Taxonomy: Classification and Nomenclature of Viruses: Ninth Report of the International Committee on Taxonomy of Viruses. (Eds. King, A. M. Q., Adams, M. J., Carstens, E. B. & Lefkowitz, E. J.) 153-162 (Elsevier/Academic Press, Amsterdam; 2012).
38. Hurtado, C. *et al.* The African swine fever virus lectin EP153R modulates the surface membrane expression of MHC class I antigens. *Arch. Virol.* **156**, 219-234 (2011).
39. Nallaparaju, K. C. *et al.* Evasion of IFN-γ signaling by *Francisella novicida* is dependent upon *Francisella* outer membrane protein C. *PLoS One* **6**, e18201 (2011).
40. Sanapala, S. *et al.* Perforin- and granzyme-mediated cytotoxic effector functions are essential for protection against *Francisella tularensis* following vaccination by the defined *F. tularensis* subsp. *novicida* Δ*fopC* vaccine strain. *Infect. Immun.* **80**, 2177-2185 (2012).
41. Gil, H. *et al.* Deletion of TolC orthologs in *Francisella tularensis* identifies roles in multidrug resistance and virulence. *Proc. Natl. Acad. Sci. U.S.A.* **103**, 12897-12902 (2006).
42. Doyle, C. R., Pan, J. A., Mena, P., Zong, W. X. & Thanassi, D. G. TolC-dependent modulation of host cell death by the *Francisella tularensis* live vaccine strain. *Infect. Immun.* **82**, 2068-2078 (2014).
43. Su, J. *et al.* Genome-wide identification of *Francisella tularensis* virulence determinants. *Infect. Immun.* **75**, 3089-3101 (2007).
44. Hickey, A. J., Hazlett, K. R., Kirimanjeswara, G. S. & Metzger, D. W. Identification of *Francisella tularensis* outer membrane protein A (FopA) as a protective antigen for tularemia. *Vaccine* **29**, 6941-6947 (2011).

45. Tempel, R., Lai, X. H., Crosa, L., Kozlowicz, B. & Heffron, F. Attenuated *Francisella novicida* transposon mutants protect mice against wild-type challenge. *Infect. Immun.* **74**, 5095-5105 (2006).
46. Chong, A. *et al.* Structure-function analysis of DipA, a *Francisella tularensis* virulence factor required for intracellular replication. *PLoS One* **8**, e67965 (2013).
47. Kraemer, P. S. *et al.* Genome-wide screen in *Francisella novicida* for genes required for pulmonary and systemic infection in mice. *Infect. Immun.* **77**, 232-244 (2009).
48. Michell, S. L. *et al.* Deletion of the *Bacillus anthracis capB* homologue in *Francisella tularensis* subspecies tularensis generates an attenuated strain that protects mice against virulent tularaemia. *J. Med. Microbiol.* **59**, 1275-1284 (2010).
49. Jia, Q. *et al.* A *Francisella tularensis* live vaccine strain (LVS) mutant with a deletion in *capB*, encoding a putative capsular biosynthesis protein, is significantly more attenuated than LVS yet induces potent protective immunity in mice against *F. tularensis* challenge. *Infect. Immun.* **78**, 4341-4355 (2010).
50. Gómez-Puertas, P. *et al.* The African swine fever virus proteins p54 and p30 are involved in two distinct steps of virus attachment and both contribute to the antibody-mediated protective immune response. *Virology* **243**, 461-471 (1998).
51. Salas, M. L. & Andrés, G. African swine fever virus morphogenesis. *Virus Res.* **173**, 29-41 (2013).
52. Alonso, C. *et al.* African swine fever virus-cell interactions: from virus entry to cell survival. *Virus Res.* **173**, 42-57 (2013).
53. Alonso, C. *et al.* African swine fever virus protein p54 interacts with the microtubular motor complex through direct binding to light-chain dynein. *J. Virol.* **75**, 9819-9827 (2001).
54. Borca, M. V. *et al.* An African swine fever virus gene with similarity to the T-lymphocyte surface antigen CD2 mediates hemadsorption. *Virology* **199**, 463-468 (1994).
55. Rodríguez, J. M., Yáñez, R. J., Almazán, F., Viñuela, E. & Rodriguez, J. F. African swine fever virus encodes a CD2 homolog responsible for the adhesion of erythrocytes to infected cells. *J. Virol.* **67**, 5312-5320 (1993).
56. Borca, M. V. *et al.* Deletion of a CD2-like gene, *8-DR*, from African swine fever virus affects viral infection in domestic swine. *J. Virol.* **72**, 2881-2889 (1998).
57. Hurtado, C. *et al.* The C-type lectin homologue gene (EP153R) of African swine fever virus inhibits apoptosis both in virus infection and in heterologous expression. *Virology* **326**, 160-170 (2004).
58. Seiler, C. Y. *et al.* DNASU plasmid and PSI:Biology-Materials repositories: resources to accelerate biological research. *Nucleic Acids Res.* **42**, D1253-1260 (2014).
59. Sykes, K. F. & Johnston, S. A. Genetic live vaccines mimic the antigenicity but not pathogenicity of live viruses. *DNA Cell Biol.* **18**, 521-531 (1999).
60. Whitlock, G. C. *et al.* Protective antigens against glanders identified by expression library immunization. *Front. Microbiol.* **2**, 227 (2011).
